# Genetic Regulation of Alternative Polyadenylation Provides Novel Insights into Molecular Mechanisms Underlying Non‐small Cell Lung Cancer

**DOI:** 10.1002/advs.202502008

**Published:** 2025-04-26

**Authors:** Meng Jin, Yongbiao Huang, Bin Li, Yuan Wang, Yan Li, Zhirui Chen, Zhe Tang, Chaofan Liu, Lei Zhang, Xianglin Yuan, Jianbo Tian, Bo Liu

**Affiliations:** ^1^ Department of Oncology Tongji Hospital Tongji Medical College Huazhong University of Science and Technology Wuhan Hubei 430030 China; ^2^ Department of Epidemiology and Biostatistics School of Public Health Wuhan University Wuhan 430071 China; ^3^ Department of Thoracic Surgery Tongji Hospital Tongji Medical College Huazhong University of Science and Technology Wuhan Hubei 430030 China; ^4^ Department of Pulmonary and Critical Care Medicine NHC Key Laboratory of Respiratory Diseases Tongji Hospital Tongji Medical College Huazhong University of Science and Technology 1095 Jiefang Ave Wuhan 430030 China; ^5^ Department of Epidemiology and Biostatistics School of Public Health Department of Gastrointestinal Oncology Zhongnan Hospital of Wuhan University TaiKang Center for Life and Medical Sciences Wuhan University Wuhan 430071 China

**Keywords:** alternative polyadenylation, apaQTLs, functional PRS, LYRM4, non‐small cell lung cancer

## Abstract

Emerging evidence emphasizes the critical role of alternative polyadenylation (APA) in posttranscriptional regulation of genes, and APA‐associated genetic variants (apaQTLs) show particular relevance for multiple disease. However, genetic regulation of APA and its role in non‐small cell lung cancer (NSCLC) risk have not been thoroughly studied. Here, by leveraging genotype and APA data from The Cancer Genome Atlas, the association between genetic variation and APA is determined in NSCLC samples. The identified apaQTLs are distinct from eQTLs and are preferentially enriched in functionally relevant characteristics, including poly(A) motifs, APA‐associated RBP binding sites, functional elements, and known NSCLC risk loci. Moreover, genes associated with apaQTLs are broadly involved in cancer‐related biological process. Of note, integration of apaQTL variants with traditional GWAS‐derived PRS is proved as a potential screening tool for NSCLC. By integrating large‐scale population and biological experiments, a functional apaQTL variant rs9606 in *LYRM4* is identified. Mechanistically, rs9606 induces aberrant APA process of *LYRM4* via allele‐specific interacting with NUDT21, which lead to increased expression of oncogene *LYRM4* and thus contribute to NSCLC risk. This study demonstrates the distinct contribution of APA‐associated genetic variants in NSCLC risk, providing critical clues and potential targets for NSCLC etiology and clinical intervention.

## Graphical abstract

Compared with the non‐risk G allele of rs9606, the risk T allele of rs9606 decreases the binding affinity of NUDT21 for LYRM4, triggering 3'UTR shortening that stabilizes *LYRM4* mRNA and elevates its expression. Increased LYRM4 expression promoted malignant phenotypes of non‐small cell lung cancer (NSCLC) cells through modulating ferroptosis, supporting population findings linking rs9606[T] to increased higher NSCLC risk.

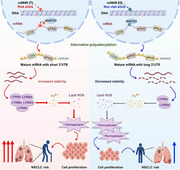

## 1 Introduction

Lung cancer remains one of the most common cancers and the leading cause of cancer‐related mortality worldwide, poses a serious threat to human health.^[^ ^1^ ^]^ Non‐small cell lung cancer (NSCLC) is the commonest form and accounts for the 85% of total lung cancers.^[^ ^2^ ^]^ It is well known that, in addition to environmental risk factors (such as tobacco smoking), genetic variants also contribute to risk for NSCLC.^[^ ^3^ ^]^ Genome‐wide association studies (GWAS) have identified numerous genetic variants associated with NSCLC risk, but identifying causal variants and elucidating their functional mechanism remains an ongoing challenge, as GWAS data cannot directly reveal why a genetic variant is pathogenic.^[^ ^4^ ^]^ To date, most of studies concerning revealing mechanism of genetic variants impacting messenger RNA abundance (expression quantitative trait loci, eQTLs), other regulatory mechanisms beyond the genetic control of mRNA expression are underexplored.^[^ ^5^ ^]^

One such underexplored mechanism is alternative polyadenylation (APA). It is estimated that nearly 70% of human genes have multiple polyadenylation sites undergo APA. By employing different poly(A) sites, APA yields transcripts with varying 3′‐untranslated region (UTR) lengths.^[^ ^6^ ^]^ Since 3′UTR harbors regulatory elements for RNA‐binding proteins (RBPs) and miRNAs, APA can regulate gene expression by altering mRNA stability, translational efficiency, or cellular localization of proteins.^[^ ^7^ ^]^ It is well‐established that APA is strikingly dysregulated in cancer.^[^ ^8^ ^]^ Aberrant APA leading to 3′UTR shortening is a common feature of multiple types of cancer and result in dysregulation of oncogenes and tumor‐suppressor genes.^[^ ^9^ ^]^ Moreover, altered expression of key APA regulators (such as NUDT21, PCF11) often shifts APA globally and disrupts biological processes involved in cancer, including cell cycle, apoptosis, and proliferation.^[^ ^10^ ^]^ Despite these observations, our knowledge of the underlying mechanisms that induce aberrant APA is still limited.

Noticeably, growing studies have established functional links between genetic variants and APA regulation.^[^ ^11^ ^]^ Genetic variants that impact APA process have been implicated in diseases including cancer.^[^ ^12^ ^]^ Examples include a genetic variation rs78378222 changes the polyadenylation signal of *TP53* thus leading to impaired 3′end processing of *TP53* mRNA and further promoting oncogenesis.^[^ ^13^ ^]^ Similarly, rs1020670 at *DNM1L* 3′UTR locus can promote aberrant APA and shorten the 3′UTR of *DNM1L*, which stabilize *DNM1L* mRNA to upregulate its expression and further lead to increased colorectal cancer risk.^[^ ^11b^ ^]^ A recent work identified ≈0.4 million genetic variants associated with APA, which contribute substantially to the molecular mechanisms underlying human diseases.^[^ ^11a^ ^]^ These findings highlight the importance of genetic regulation of APA as a molecular mechanism for disease risk. However, to date, large‐scale apaQTL studies conducted in NSCLC have been sparse, the mechanisms of genetic regulation of APA and their associations with NSCLC risk have not been systematically studied.

To fill this gap, by integrating genotype and APA phenotype data from TCGA, we systematically investigate the genetic regulation of APA in NSCLC tissues. We identified 117 161 significant genetic variants that are associated with APA (apaQTL variants) and 2037 apaQTL‐associated genes. Then, we comprehensively characterized the functional properties of these apaQTLs and the corresponding target genes. Furthermore, we used whole‐genome sequencing genotype data derived from 13 835 NSCLC patients and 13 835 controls to assess the effect of apaQTLs on NSCLC risk. We generated PRS score by using apaQTL variants and known NSCLC risk variants, and evaluated its utility and effectiveness in predicting NSCLC risk in multiple cohorts. By integrating large‐scale population and biological experiments, we confidently identified a functional apaQTL variant rs9606 in *LYRM4* contributes to NSCLC risk through genetic regulation of APA. Collectively, our findings advance understanding of molecular mechanism linking genetic variants to NSCLC risk and represents a valuable resource for identification of novel therapeutic targets.

## 2 Results

### 2.1 Identification and Characterization of apaQTLs in Nonsmall Cell Lung Cancer

**Figure 1:**
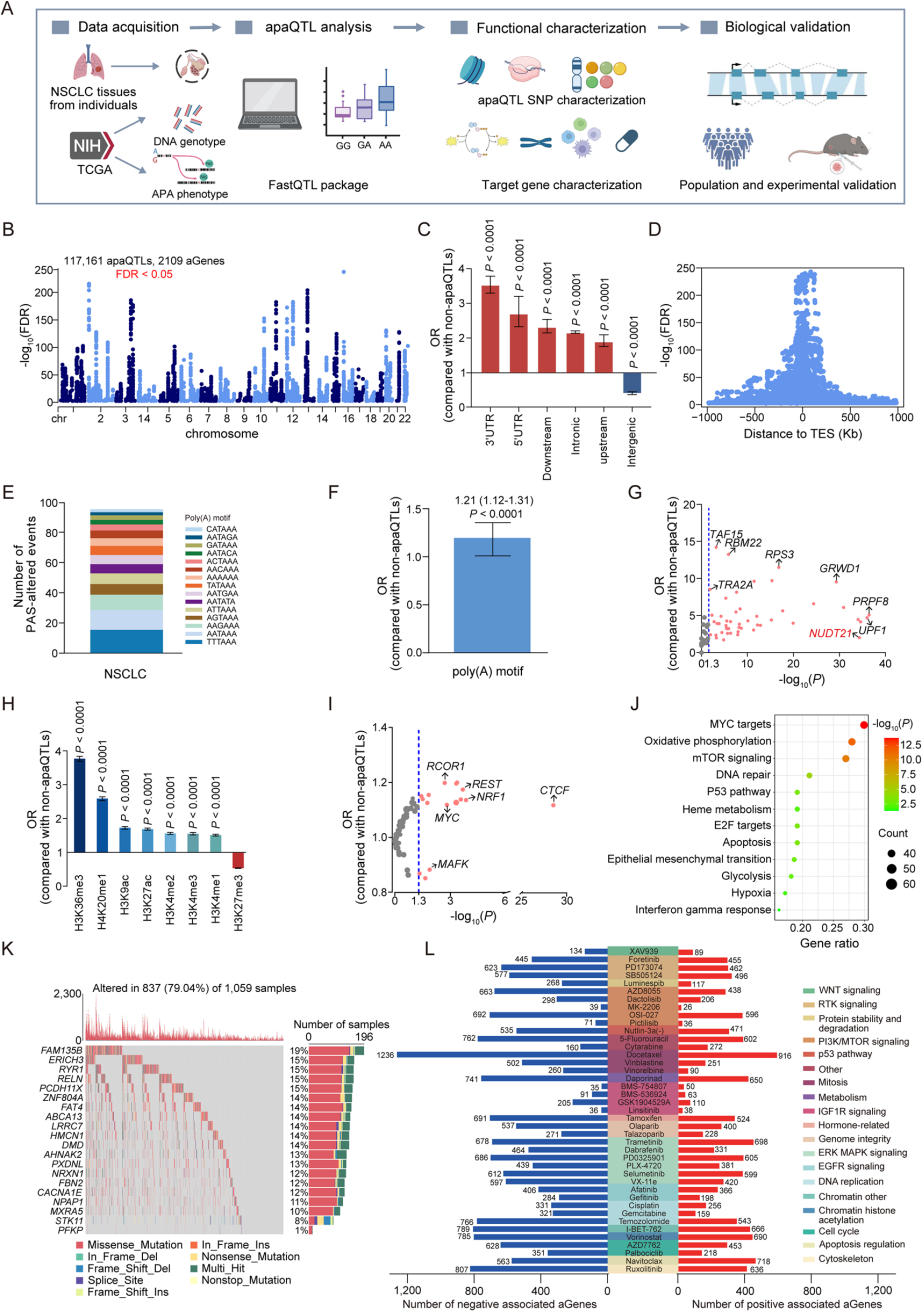
Identification and functional characterization of apaQTLs in NSCLC. A) An overview of study design and apaQTL analysis workflow. Integration APA phenotype and genotype data of 1010 NSCLC samples from TCGA, a genome‐wide apaQTL analysis was performed to identify APA‐associated variants in NSCLC. We then performed functional annotation of apaQTLs and the its corresponding target genes (aGenes). The biological function of NSCLC‐associated apaQTL variants rs9606 was further investigated by and population validation and experimental approach. B) Manhattan plots displaying apaQTL analysis result in NSCLC. The ‐log_10_(FDR) for each variant is plotted according to chromosome position. FDR < 0.05 was considered statistically significant. C) Enrichment of apaQTL SNPs in each genomic region. *P*‐values were calculated by two‐tailed Fisher's exact test. Error Bar indicate 95% confidence intervals (CIs). D) Distance of apaQTLs from their target genes (aGenes). SNPs were plotted according to its distance to the aGene and statistical significance for association with APA event. TES, transcription end site. E) Overview of PAS motifs altered by apaQTLs in NSCLC. F) Enrichment of apaQTLs in PAS motifs, compared with non‐apaQTLs. *P*‐values were calculated by two‐tailed Fisher's exact test. Error bar, 95% CI. G) Enrichment of apaQTLs in binding sites for individual RBPs. Blue dashed line: *P* < 0.05. H) Enrichment analyses of apaQTLs in histone modification markers annotated by ENCODE, compared with non‐apaQTLs. *P*‐values were calculated by two‐tailed Fisher's exact test. Error bars, 95% CIs. I) Enrichment of apaQTLs in TF‐binding sites of individual TFs. Blue dashed line: *P* < 0.05. J) KEGG pathway enrichments for aGenes. K) Somatic copy‐number alteration (SCNA) pattern in NSCLC tumor samples. The top 20 most frequently mutated genes are shown in each sample, and the mutation subtypes are indicated by color. L) Significant aGene‐drug pairs identified in Genomics of Drug Sensitivity in Cancer (GDSC) dataset. Red and blue bars denote positive and negative associations, respectively.

As shown in **Figure** **1**A, we performed a genome‐wide apaQTL analysis in NSCLC, using percentage of distal poly(A) site usage index (PDUI) of APA events, genotype and clinical characteristics data of 1010 NSCLC patients from TCGA. After quality control filtering (Experimental Section; and Figure S1, Supporting Information), we included genetic data of 4022558 SNPs and 4243 APA events for further analysis. Adjusting for known and inferred covariates, we totally identified 117 161 significant apaQTL SNPs in TCGA NSCLC tissues (FDR < 0.05, Figure 1B). To characterize genomic features of identified apaQTL SNPs, we generated a set of control SNPs from the genome with MAF and LD scores matched with apaQTL SNPs. We functionally annotated apaQTL SNPs and control SNPs according to categories defined by SnpEff (Figure S2, Supporting Information). The results showed that compared with control SNPs, apaQTL SNPs were significantly enriched in 3′UTR [OR (95% CI), 3.53 (3.30–3.78); *P* < 0.0001] and underrepresented among intergenic regions [OR (95% CI), 0.377 (0.371–0.383); *P* < 0.0001; Figure 1C]. Consistent with previous studies,^[^ ^11a^ ^]^ when we analyzed the relative position of apaQTL SNPs across their corresponding genes (aGenes), we found that apaQTL SNPs clustered around transcription end sites (Figure 1D).

### 2.2 apaQTLs are Enriched in Poly(A) Motifs, APA‐Associated RBP Binding Sites, and Functional Elements

Next, we explored the potential mechanisms through which apaQTLs regulate APA events. Since previous studies have shown that apaQTLs may alter the polyadenylation signal (PAS),^[^ ^14^ ^]^ we examined the prevalence of PAS‐altering apaQTLs in NSCLC and extracted significant apaQTL variants within 50 bp upstream of annotated poly(A) sites from Poly(A) database.^[^ ^15^ ^]^ By conducting motif searches based on 15 common PAS motif, we identified 94 apaQTL variants that may either alter canonical PAS or change other PAS motifs in NSCLC (Figure 1E). As expected, we also found apaQTLs were significant enriched among poly(A) motifs when compared with control SNPs [OR (95% CI), 1.21 (1.12–1.31); *P* < 0.0001; Figure 1F]. Taken together, these findings suggest that a fraction of apaQTLs affect APA events by altering poly(A) motifs.

The alteration of poly(A) motifs only explained a fraction of apaQTLs, implying that other mechanisms may also play a role. Given the critical role of RNA binding proteins (RBPs) in regulating APA process, we reasoned that if apaQTLs affect APA by impacting RBP binding sites, which was important for transcript 3′‐end processing. To test this hypothesis, we further performed enrichment analysis of apaQTLs in RBP binding sites by analyzing crosslinking and immunoprecipitation followed by sequencing (CLIP‐seq) data from the ENCODE.^[^ ^16^ ^]^ We tested apaQTLs enrichment for each RBP, and found that 55 RBPs exhibited significant enrichment among the 74 examined RBPs (Figure 1G). Among them, several APA regulators and splicing factors were identified, and a master APA mediator NUDT21 was also identified.^[^ ^10a^ ^]^

Recent studies suggest that transcription can influence gene APA process.^[^ ^17^ ^]^ We performed enrichment analyses by using ChIP‐seq data from ENCODE portal. We found that apaQTLs were significantly enriched in histone marks of enhancers (H3K27ac, H3K4me1), promoters (H3K4me3), and active transcription (H3K36me3, H4K20me1, H3K9ac, H3K4me2), whereas they were underrepresented among repressive epigenetic marker (H3K27me3) (Figure 1H). We further performed enrichment analysis of apaQTLs within 59 TF binding sites. The result showed that 18 TFs significantly enriched with apaQTLs (Figure 1I). Interestingly, the apaQTL enrichment with the most significant *P* values correspond to TFs involved in APA regulation process (CTCF gene).^[^ ^18^ ^]^ Overall, the data suggest that apaQTLs may have a unique epigenomic regulation mechanism and that transcription and polyadenylation are tightly linked.

### 2.3 apaQTLs Targeted Genes Play Crucial Roles in Cancer Development and Therapy

According to above apaQTL analysis, we identified a total of 2037 genes and 2109 associated APA events. To further investigate the functional roles of apaQTLs targeted genes (aGenes) in cancer development and therapy, we first performed pathway enrichment analysis of aGenes. Kyoto Encyclopedia of Genes and Genomes (KEGG) analysis showed that aGenes were enriched in cancer‐related processes, including P53 pathway, oxidative phosphorylation, cell apoptosis, and DNA repair pathway (Figure 1J). As somatic copy‐number alteration (SCNA) may disrupt expression levels of cancer genes and influence drug sensitivity, we next assessed SCNA in NSCLC based on the data from TCGA. By integrating aGenes that ranked top 20 among somatic mutation genes for analysis, we observed that aGenes were subject to deleterious missense mutations in majority of NSCLC patients (79.04%) (Figure 1K). Considering the emerging role of immune cell in modulating cancer development, we further assessed their association with aGene expression, in which immune cell infiltration data were quantified using the TIMER and EPIC algorithms. The results showed that several aGenes are associated with specific immune cell subsets, including CD8^+^ T cell, CD4^+^ T cell, and macrophage cell (Figures S3 and S4, Supporting Information).

To understand the effects of aGenes on drug response, we performed a systematic pharmacogenomic analyses by using Genomics of Drug Sensitivity in Cancer (GDSC) data,^[^ ^19^ ^]^ and identified a total of 34 902 significant associated aGene‐drug pairs (Figure 1L). We focused on drugs highly associated with aGenes and their pharmaceutical target. Noticeably, the top drug category is that drugs targeting ERK/MAPK signaling pathway including Trametinib and Dabrafenib (Figure S5, Supporting Information), which are approved by FDA for use in combination for the treatment of NSCLC.^[^ ^20^ ^]^ Collectively, the results of analyses above suggest the unique role of apaQTLs targeted genes in cancer development and treatment.

### 2.4 Distinct Contribution of apaQTLs to NSCLC Risk

**Figure 2:**
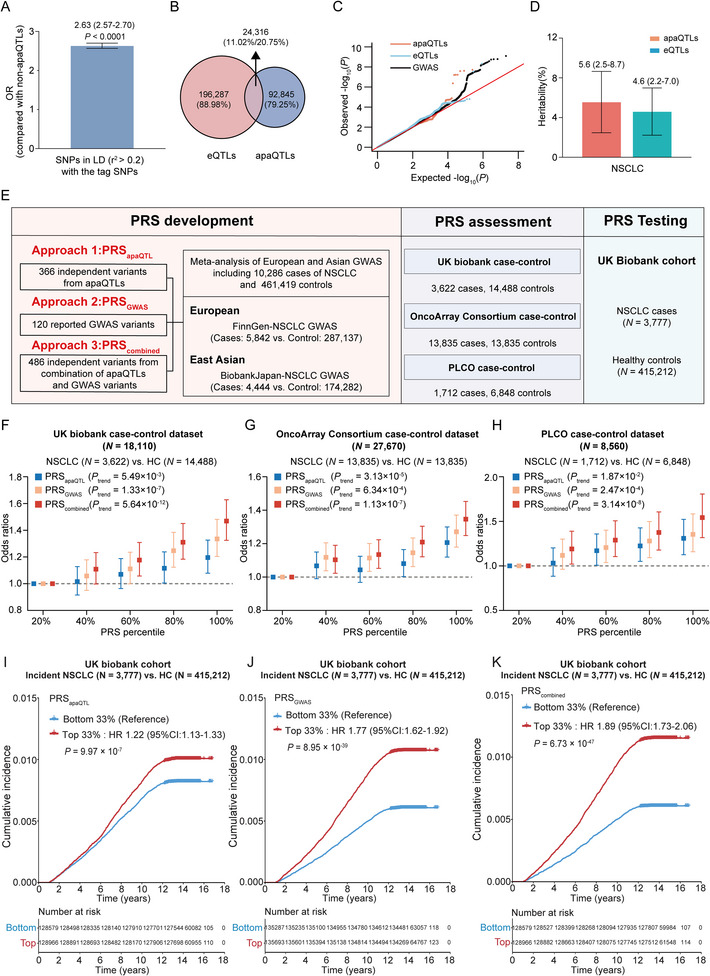
Distinct role of apaQTLs in interpreting NSCLC GWAS variants and risk prediction. A) Enrichment of apaQTLs among GWAS loci by the two‐tailed Fisher's exact test comparing apaQTLs to control non‐apaQTLs. Error bar, 95% CI. B) Overlap between apaQTLs and eQTLs in NSCLC. C) Quantile–quantile (QQ) plot showing the *P*‐values of NSCLC GWAS SNPs, which were binary annotated by apaQTLs and eQTLs with FDR < 0.05. D) Proportion of GWAS trait heritability explained by apaQTLs and eQTLs. The bars indicate standard errors. E) Overview of PRS models development, assessment, and testing strategy. F–H) Odds ratios (ORs) for NSCLC risk of each PRS group by comparing with those in bottom 20% under three PRS models in UKB case‐control set F), OncoArray Consortium case‐control set G), and PLCO case‐control set H). PRS_apaQTL_ indicates the PRS derived from independent apaQTLs, PRS_GWAS_ indicates the PRS derived from known NSCLC GWAS variants, PRS_Combined_ indicates the PRS derived from combination of apaQTLs and NSCLC GWAS variants. Error bars, 95% CIs. I) Cumulative incidences of top 33% and bottom 33% of PRS_apaQTL_ in UK Biobank cohort. Hazard ratios and the 95% confidence intervals derived from Cox proportional hazards regression model. J) Cumulative incidences of top 33% and bottom 33% of PRS_GWAS_ in UK Biobank cohort. Hazard ratios and the 95% confidence intervals derived from Cox proportional hazards regression model. K) Cumulative incidences of top 33% and bottom 33% of PRS_Combined_ in UK Biobank cohort. Hazard ratios and the 95% confidence intervals derived from Cox proportional hazards regression model.

As genetic variation may contribute to disease risk through APA, we next tested whether the identified apaQTL SNPs contribute to NSCLC risk. By integrating GWAS summary statistics from previous work (Methods, Supporting Information), we evaluated the enrichment of apaQTLs within NSCLC GWAS loci. Of note, apaQTL SNPs were significantly enriched in NSCLC GWAS loci compared with control SNPs [OR (95% CI), 2.63 (2.57–2.70); *P* < 0.0001; **Figure** **2**A]. Additionally, by conducting colocalization analysis, we totally found 2058 apaQTLs colocalizing with 12 NSCLC GWAS signals. As an example, apaQTL SNP rs8042489 was identified to colocalize with a NSCLC GWAS signal in the 15q25.1 loci (Figure S6A, Supporting Information), which was significantly associated with 3′UTR usage in *TBC1D2B* (Figure S6B, Supporting Information). Recent studies demonstrated that *TBC1D2B* functions as a *Rab22*‐binding protein and promotes lung cancer oncogenesis.^[^ ^21^ ^]^ Moreover, rs8042489 conferred a significant genetic predisposition to NSCLC (Figure S6C, Supporting Information). These results suggest that apaQTL variant rs8042489 might affect *TBC1D2B* APA process, thus contributing to NSCLC risk. To further understand the contribution of apaQTLs to NSCLC risk, eQTLs, a fundamental mechanism of gene regulation, were included for comparison. To conduct comparison between apaQTLs and eQTLs, we downloaded NSCLC eQTL data from PancanQTL database (http://bioinfo.life.hust.edu.cn/PancanQTL/).^[^ ^22^ ^]^ Then, we calculated the overlap proportion of each two of them. The observed overlap between apaQTLs and eQTLs was moderate, with only 20.75% of apaQTL SNPs overlapping with eQTL SNPs (Figure 2B). These results are consistent with observations from other studies,^[^ ^11^, ^23^ ^]^ suggesting that apaQTLs exert their functional roles independent of regulation of gene expression. Furthermore, both apaQTLs and eQTLs showed an excess of low *P* values in NSCLC GWASs, and apaQTLs appeared to have larger magnitude effects than eQTLs (Figure 2C). Consistently, partitioned heritability estimation using linkage disequilibrium (LD) score regression revealed higher enrichment of NSCLC heritability in apaQTLs than in eQTLs (Figure 2D). We also validate these results in the Finnish biobank (FinnGen) and found that apaQTLs account for 5.23% of cancer heritability, which is higher than that of eQTLs (4.15%) (Figure S7, Supporting Information). Additionally, we removed the apaQTLs that overlapped with eQTLs and subsequently conducted QQ plot analysis and heritability estimation. The results indicate that apaQTLs remain a significant ability in cancer heritability estimation (Figure S8, Supporting Information). Collectively, our findings suggest that most apaQTLs are distinct from eQTLs, demonstrating the distinct contribution of apaQTLs to NSCLC risk.

### 2.5 Polygenic Risk Scores from apaQTLs Improve Risk Prediction for NSCLC

Previous studies have shown that polygenic risk score (PRS) derived from functional genetic variants can serve as efficient tool for disease risk assessment. To investigate the clinical utility of NSCLC apaQTLs in predicting NSCLC risk, we constructed PRS models by using genetic variants from three different sets, including 366 independent apaQTL variants after LD clumping (*r* ^2^>0.2, PRS_apaQTL_), 120 known NSCLC risk variants (PRS_GWAS_), and 486 independent variants (PRS_Combined_) from a combination of apaQTL and GWAS variants (Figure 2E). We then compared the predictive performance of each of these PRSs in three independent NSCLC study groups from UK biobank, OncoArray Consortium Lung Cancer Studies, and PLCO. Initially, we performed the NSCLC risk stratification in UK biobank dataset, including 3622 cases and 14 488 controls. We found that apaQTLs‐derived PRS exhibited a moderate capability of NSCLC risk prediction across each grade of genetic risk, with high genetic risk (in the top 20% PRS) had a 1.20‐fold increased NSCLC risk of compared to those with low genetic risk (in the bottom 10% PRS; *P*_trend_ = 5.49×10^−3^, Figure 2F). Importantly, PRS_combined_ outperformed the other two PRS models by the values of OR (OR = 1.47, 95%CI = 1.32–1.63; *P*_trend_ = 5.64×10^−12^, Figure 2F), indicating that apaQTL variants can be used to improve NSCLC risk stratification. A similar trend was observed in two external validation sets from OncoArray (OR [95%CI] = 1.35[1.25–1.45], *P*_trend_ = 1.13×10^−7^, Figure 2G) and PLCO (OR [95%CI] = 1.54[1.32–1.81], *P*_trend_ = 3.14×10^−8^, Figure 2H).

We further evaluated its effectiveness in risk stratification in a prospective UK biobank cohort consisting of 418 989 individuals, of whom 3777 developed NSCLC during follow‐up. The apparently separate predictive cumulative incidence curves were observed in individuals at low (bottom 33% of PRS) and high genetic risk (top 33% of PRS; Figure 2I–K). Consistent with the findings in case‐control dataset, individuals with high apaQTLs‐derived PRS had higher risk of incident NSCLC than individuals with low genetic risk (HR = 1.22, 95% CI = 1.13–1.33, *P* = 9.97×10^−7^, Figure 2I). Of note, PRS_Combined_ still showed the optimal discriminatory ability to stratify individual NSCLC risk (Figure 2K). These results highlight the potential clinical utility of PRS_apaQTL_ to improve NSCLC risk prediction by combining with traditional GWAS‐derived PRS. Taken together, our findings suggest that integration of apaQTL variants with traditional GWAS‐derived PRS improves genetic risk prediction for NSCLC.

### 2.6 apaQTL Variant rs9606 is Significantly Associated with NSCLC Risk

**Figure 3:**
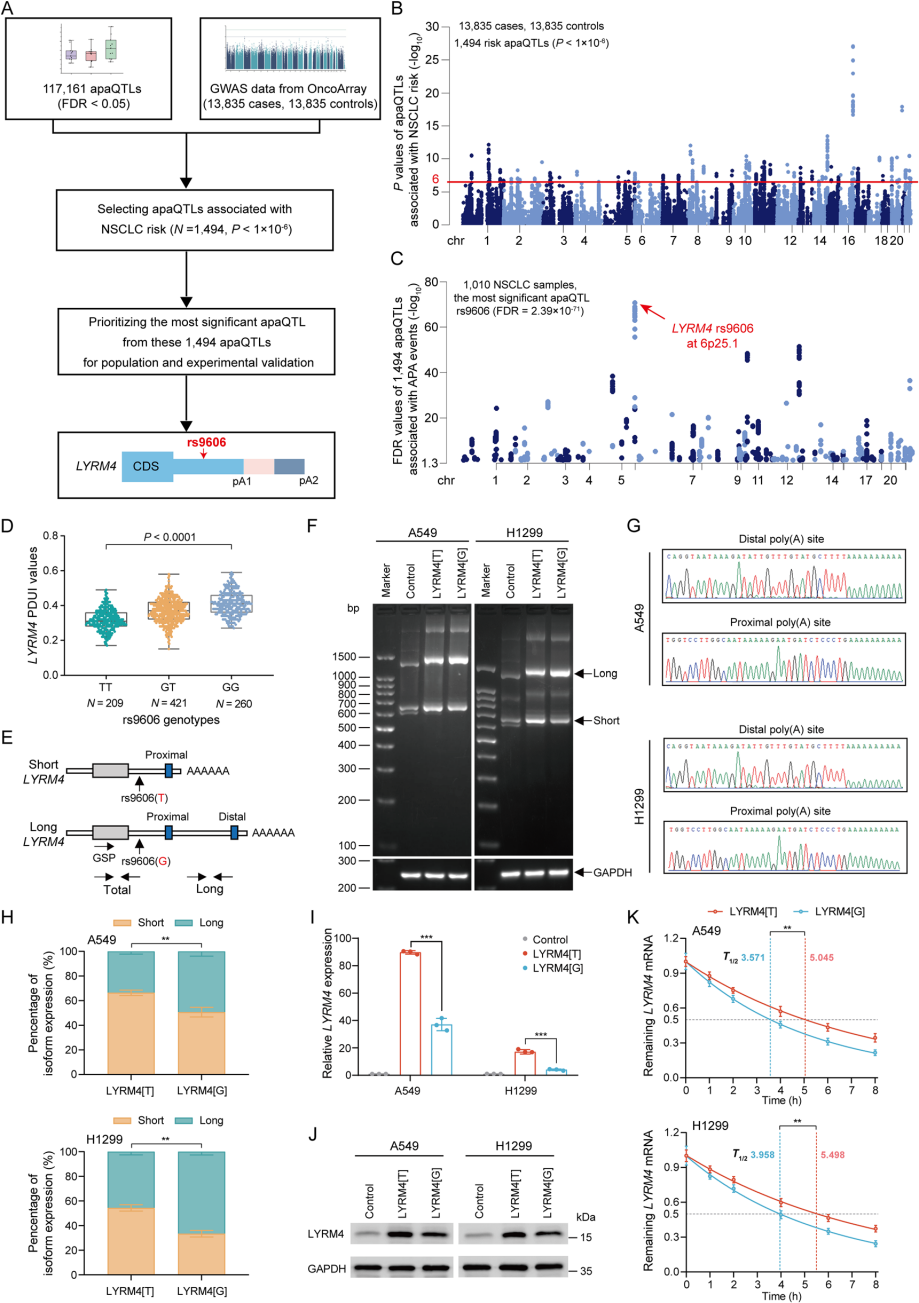
The *LYRM4* rs9606 variant as the putative functional apaQTL link alternative polyadenylation to NSCLC risk. A) Flowchart of identification and validation of functional risk apaQTLs in NSCLC. B) Manhattan plot shows ‐log_10_(*P* value) for associations of apaQTLs with NSCLC risk estimated by OncoArray GWAS data consisting of 13 835 cases and 13 835 controls. The red line denotes the threshold of significance (*P* < 1×10^−6^). C) Manhattan plot depicted the ‐log_10_(FDR value) of 1494 apaQTLs that associated with NSCLC risk. The most significant apaQTL *LYRM4* rs9606 was annotated with red arrow. D) Box plot showing that apaQTL rs9606 was significantly associated with *LYRM4* 3′UTR usage for each genotype in TCGA NSCLC samples. *P* value was calculated by One‐Way ANOVA. E) Schematic depicts *LYRM4* with short and long 3′UTR. F) 3′‐RACE were performed to determine the effect of *LYRM4* rs9606 genotypes on *LYRM4* 3′UTR usage in A549 and H1299 cells. G) Sanger sequencing confirmed the precise polyadenylation sites within *LYRM4* transcript. H) qRT‐PCR validated the effect of *LYRM4* rs9606 genotypes on *LYRM4* 3′UTR usage in A549 and H1299 cells. I,J) The *LYRM4* mRNA I) and protein expression J) levels were detected by qRT‐PCR and western blotting in NSCLC cells overexpressing control vector, LYRM4[T], and LYRM4[G] vector. K) The effect of *LYRM4* rs9606 genotypes on *LYRM4* mRNA stability were determined by qRT‐PCR in A549 and H1299 cells after treating with actinomycin D (5 µg mL^−1^) at indicated time points. For panels (H–K), the data were shown as mean ± S.D. from three independent experiments. *P* values were calculated using Student's *t*‐test. **, *P* < 0.01; ***, *P* < 0.001.

To identify the casual apaQTL variants that contribute to NSCLC risk by modulating APA events, we integrated 117 161 apaQTLs (FDR < 0.05) with NSCLC GWAS data from OncoArray Consortium lung cancer studies (13 835 cases and 13 835 controls, **Figure** **3**A). The demographic characteristics of the GWAS dataset were summarized in Table S1 (Supporting Information). To identify more functional apaQTL variants, we applied a significance level of *P* < 1×10^−6^ which is widely used in previous studies.^[^ ^24^ ^]^ A total of 1494 apaQTLs were identified to be associated with NSCLC risk (Figure 3B). Among these risk apaQTL variants, rs9609 showed the most significant association with APA events (Figure 3C). rs9606 was located in the 3′UTR of *LYRM4* and showed strong association with alternative 3′UTR of *LYRM4* mRNA, implying its potential role in APA regulation. T allele of rs9606 was also revealed to be associated with increased risk of NSCLC in OncoArray NSCLC samples (OR = 1.09; 95%CI, 1.06–1.13; *P* = 8.77×10^−8^; **Table** **1**). Thus, we focused on apaQTL rs9606 for further investigation.

**Table 1:** The association of rs9606 with NSCLC risk in European and Chinese case‐control set (*N* = 68 358).

	OncoArray Consortium set [*N* = 27 670]			UK biobank set [*N* = 18 110]			PLCO set [*N* = 8560]			Combined set [*N* = 54 340]			
Genotypes/	Case/Control	OR (95%CI)^a)^	P^b)^	Case/Control	OR (95%CI)^a)^	P^b)^	Case/Control	OR (95%CI)^a)^	P^b)^	Case/Control	OR (95%CI)^a)^	P^b)^	MAF
rs9606													
GG	3670/3937	1.00(Ref)		922/4157	1.00(Ref)		650/2795	1.00(Ref)		5242/10889	1.00(Ref)		0.465
GT	6431/6558	1.05(0.99–1.11)	8.60×10^−2^	1767/7091	1.12(1.03–1.23)	1.12×10^−2^	771/3250	1.02(0.91–1.15)	7.22×10^−1^	8969/16899	1.10(1.05–1.15)	1.19×10^−5^	
TT	3734/3340	1.20(1.12–1.28)	6.95×10^−8^	933/3240	1.30(1.17–1.44)	6.11×10^−7^	291/803	1.56(1.33–1.83)	3.55×10^−8^	4958/7383	1.37(1.30–1.43)	3.32×10^−35^	
Dominant		1.10(1.04–1.16)	4.58×10^−4^		1.18(1.08–1.28)	1.28×10^−4^		1.13(1.01–1.26)	2.97×10^−2^		1.18(1.13–1.23)	1.80×10^−16^	
Recessive		1.16(1.10–1.22)	9.75×10^−8^		1.20(1.11–1.31)	1.50×10^−5^		1.55(1.34–1.79)	4.98×10^−9^		1.29(1.24–1.34)	1.32×10^−32^	
Additive		1.09(1.06–1.13)	8.77×10^−8^		1.14(1.08–1.20)	6.65×10^−7^		1.20(1.11–1.29)	7.19×10^−6^		1.17(1.14–1.19)	6.09×10^−34^	
Genotypes/ rs9606	Independent I set (*N* = 3400)			Independent II set (*N* = 5618)			Independent III set (*N* = 5000)			Combined set (*N* = 14018)			
	Case/Control	OR (95%CI)^a)^	P^c)^	Case/Control	OR (95%CI)^a)^	P^c)^	Case/Control	OR(95%CI)^a)^	P^c)^	Case/Control	OR(95%CI)^a)^	P^c)^	MAF
GG	1024/1053	1.00(Ref)		1675/1752	1.00(Ref)		1481/1575	1.00(Ref)		4180/4380	1.00(Ref)		0.205
GT	630/617	1.37(1.12–1.67)	2.03×10^−3^	1068/1006	1.28(1.14–1.44)	3.93×10^−5^	960/884	1.20(1.06–1.37)	4.89×10^−3^	2658/2507	1.18(1.10–1.27)	3.79×10^−6^	
TT	46/30	1.64(1.01–2.67)	4.52×10^−2^	66/51	1.50(1.03–2.18)	3.66×10^−2^	59/41	1.62(1.07–2.46)	2.29×10^−2^	171/122	1.46(1.15–1.86)	1.79×10^−3^	
Dominant		1.39(1.14–1.69)	9.59×10^−4^		1.29(1.15–1.45)	1.46×10^−5^		1.22(1.07–1.38)	2.24×10^−3^		1.20(1.12–1.28)	5.27×10^−7^	
Recessive		1.40(0.87–2.25)	1.66×10^−1^		1.36(0.93–1.97)	1.12×10^−1^		1.47(0.98–2.22)	6.51×10^−2^		1.02(0.96–1.09)	5.03×10^−1^	
Additive		1.33(1.13–1.58)	8.14×10^−4^		1.25(1.14–1.41)	1.16×10^−5^		1.22(1.09–1.40)	8.65×10^−4^		1.19(1.12–1.27)	1.21×10^−7^	

^a)^ ORs and 95% CIs calculation were conducted under assumption that variant alleles were risk alleles;

^b)^ All *P* values were calculated by unconditional logistic regression model after adjusting for gender and age group;

^c)^ All *P* values were calculated by unconditional logistic regression model after adjusting for gender, age group, smoking status and drinking status; Abbreviations: Ref, reference; OR, odds ratio; CI, confidence interval.

We adopted NSCLC genotype data from the UK biobank and PLCO cohort to strengthen the population epidemiological findings in OncoArray NSCLC cohorts. The demographic characteristics were listed in Tables S2 and S3 (Supporting Information). After combined analysis, the result showed that the association between apaQTL and NSCLC risk remained significant, with the T allele having an OR for NSCLC risk of 1.17 (95% CI, 1.14–1.19; *P* = 6.09×10^−34^) in the addictive model (Table 1). Furthermore, we validate the association between rs9606 and NSCLC risk in three independent Chinese cohorts. A total of 1700 NSCLC cases and 1700 healthy controls were included in phase I, 2809 cases and 2809 controls were included in phase II, and 2500 cases and 2500 controls were included in phase III. The demographic characteristics of the study subjects were summarized in Table S4 (Supporting Information). Significant associations were verified in both phases. Compared with rs9606 GG genotype, individuals with TT genotype had higher risk of NSCLC. Combined analysis of two phases showed that rs9606 TT genotype was still associated with increased risk of NSCLC, with odds ratio of 1.19 (95% CI, 1.12–1.27; *P* = 1.21×10^−7^) under the addictive model (Table 1).

### 2.7 Risk T allele of rs9606 Facilitates *LYRM4* Expression by Regulating APA Process

To examine the functional significance of apaQTL variant rs9606 contributing to APA process in NSCLC, we first analyzed the association between rs9606 genotypes and the alternative polyadenylation of *LYRM4*. Our analysis revealed that apaQTL variant rs9606 was significantly associated with alternative 3′UTR of *LYRM4* mRNA, implying its potential role in APA regulation (Figure 3D). To validate the effect of rs9606 on APA, we performed qualitative 3′Rapid Amplification of cDNA Ends (3′RACE) and quantitative polymerase chain reaction (qPCR) analyses of changes of *LYRM4* 3′UTR length in NSCLC cell lines (Figure 3E). In both A549 and H1299 cell lines, we found that overexpression of LYRM4 containing risk T allele of rs9606 (LYRM4[T]) increased level of *LYRM4* short 3′UTR isoform, compared with that of LYRM4 containing the nonrisk G allele of rs9606 (LYRM4[G]) and control vector (Figure 3F–H). These results suggest that risk T allele of rs9606 causes *LYRM4* 3′UTR shortening by APA.

As alterations of 3′UTR length by APA may deregulate expression of oncogenes, we assessed if risk T allele of rs9606 can modulate *LYRM4* expression. As expected, we found that overexpression of rs9606‐T allele resulted in increased mRNA and protein expression levels of *LYRM4*, compared with that of rs9606‐G allele and control vector (Figure 3I,J). To further explore the mechanism of rs9606 modulate LYRM4 expression, we first assessed *LYRM4* mRNA decay rate at multiple indicated timepoints following Actinomycin D treatment. qRT‐PCR of *LYRM4* mRNA found that overexpression of rs9606‐T allele significantly increased the stability of *LYRM4* mRNA (Figure 3K). Moreover, we determined the direct effect of changes of LYRM4 3′UTR length on its mRNA stability. The result showed that overexpression of the LYRM4 with short 3′UTR exhibited a significantly slower LYRM4 mRNA decay rate than that overexpression of LYRM4 with long 3′UTR (Figure S9, Supporting Information). Then, we performed dual luciferase reporter assays using reporter plasmids with short or long 3′UTR of *LYRM4*. We mutated the proximal poly(A) signal sequence of the plasmid containing long *LYRM4* 3′UTR to ensure the only dPAS is used (Figure S10A, Supporting Information). Notably, overexpression of plasmids with short 3′UTR showed significantly higher luciferase activity than those with long 3′UTR, suggesting 3′UTR shortening affect translation process (Figure S10B, Supporting Information). To further investigate whether the rs9606 can influence the *LYRM4* expression by directly regulating gene transcription, in addition to the APA process, we constructed luciferase vectors containing either the rs9606‐G or rs9606‐T alleles and transfected them into A549 and H1299 cell lines. Notably, we found that transfection of the vector containing the rs9606‐T allele did not affect luciferase activities in the cells compared to the vector containing the rs9606‐G allele (Figure S11, Supporting Information). Taken together, these findings demonstrate that apaQTL rs9606 function as a causal variant modulating alternative polyadenylation of *LYRM4*, leading to dysregulation of *LYRM4* by affecting mRNA stability and translation activity.

### 2.8 RNA Binding Protein NUDT21 Preferentially Bind to Nonrisk G allele of rs9606 at LYRM4

**Figure 4:**
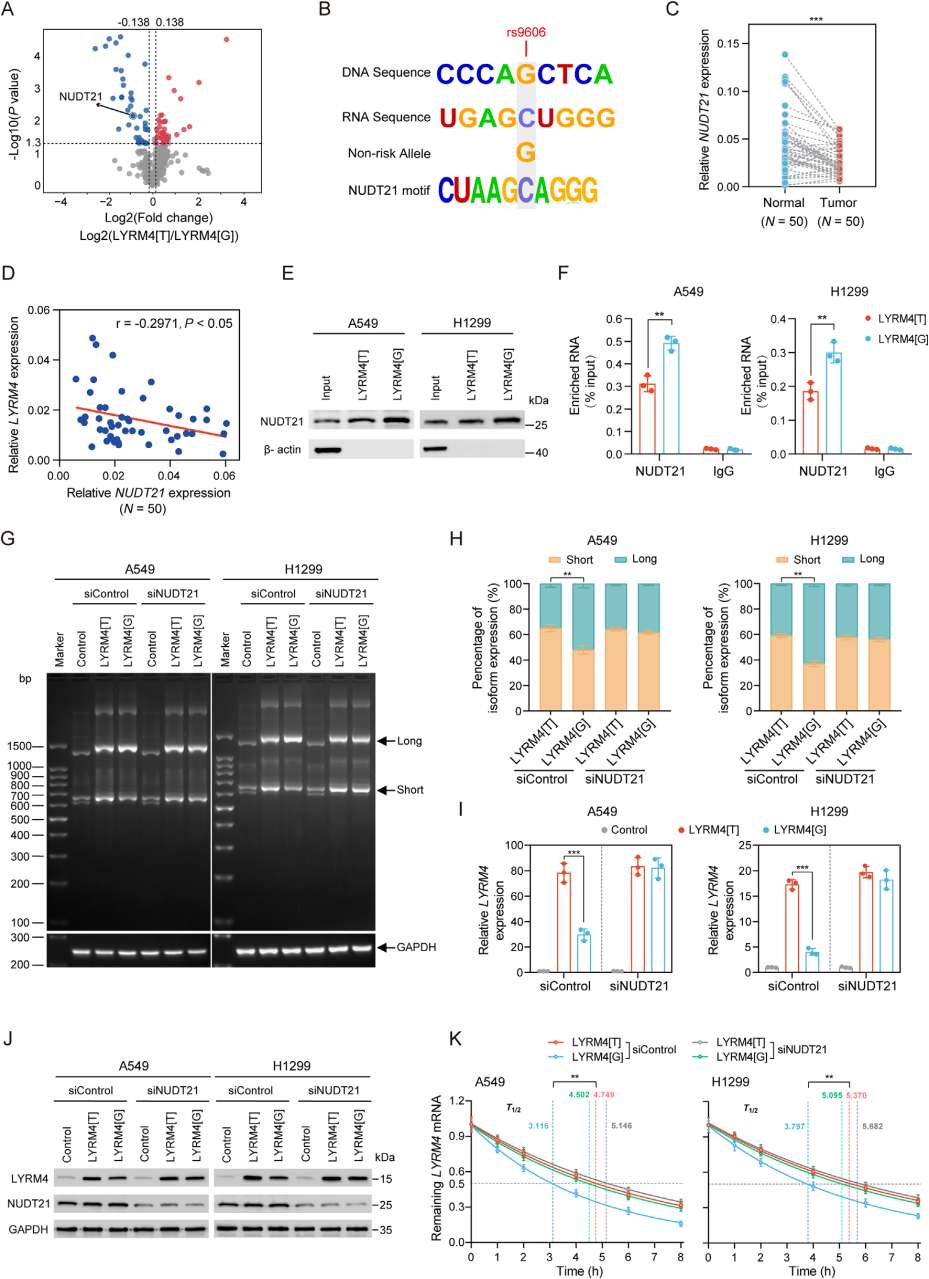
RBP NUDT21 mediates the process of *LYRM4* rs9606 variant regulating *LYRM4* APA process. A) Mass spectrometry screening for RNA binding proteins (RBPs) that differentially bound to rs9606‐G and rs9606‐T probes. Horizontal line indicates the *P* < 0.05 cutoff. Vertical lines mark the fold change > 1.1 or < 0.9. B) Predicted preferential binding of NUDT21 to nonrisk allele G of rs9606. C) The expression of *NUDT21* in our own NSCLC tissues and paired adjacent normal tissues. *P‐*value was calculated by Wilcoxon rank‐sum test. D) The correlation between *NUDT21* expression and *LYRM4* expression in our own NSCLC tissues. *P*‐value and r‐value were calculated by Spearman correlation analysis. E) The candidate RBP NUDT21 identified from proteomic screening was confirmed by RNA pull down followed by western blot analysis. F) RIP assay followed by qRT‐PCR analysis suggested the preferential binding of NUDT21 to the *LYRM4* rs9606[G] allele. Results were normalized to input from three independent experiments. IgG used as negative control here. G) NSCLC cells were seeded on plates after transfection with siRNAs targeting NUDT21 or the siControl. Then the cells were transfected with LYRM4[G], LYRM4[T], or the control vector. The difference of *LYRM4* 3′UTR usage between *LYRM4* rs9606 genotypes upon *NUDT21* knockdown were detected by 3′‐RACE assays. H) Quantification of long and short *LYRM4* 3′UTRs by qRT‐PCR in NSCLC cells. Data were presented as the mean ± SD from three independent experiments. **P < 0.01 were calculated using Student's *t*‐test. I,J) The effects of *NUDT21* knockdown on LYRM4 expression were detected by qRT‐PCR I) and western blot assays J) in cells transfected with LYRM4[G], LYRM4[T], or the control vector. Data were presented as the mean ± SD from three independent experiments. ***P < 0.001 were calculated using Student's *t*‐test. K) The effects of *NUDT21* knockdown on *LYRM4* mRNA stability were detected by qRT‐PCR in NSCLC cells after actinomycin D (5 µg mL^−1^) treatment. Data were presented as the mean ± SD from three independent experiments. ***P < 0.001 were calculated using Student's *t*‐test.

Having demonstrated apaQTL rs9606 regulates *LYRM4* expression through driving aberrant APA process, we next sought to investigate the underlying mechanism. Given that apaQTL variants are thought to modulate APA process by altering RBP binding, we examined whether rs9606 directly alters RBP binding. RNA pull‐down plus mass spectrometry (MS) assays revealed several candidate RBPs involved in APA process (**Figure** **4**A), in which NUDT21 had been reported as a critical APA regulator.^[^ ^10^, ^25^ ^]^ Further motif analysis using online CLIP‐Seq database (ENCORI platform) showed that rs9606 overlaps with predicted binding motif of NUDT21, and NUDT21 preferentially bind to nonrisk G allele of rs9606 (Figure 4B). Moreover, *NUDT21* expression was lower in NSCLC tissues compared to the adjacent normal tissues, and *NUDT21* expression was negatively correlated with *LYRM4* in our own NSCLC tissues (Figure 4C,D). Furthermore, we investigated the effect of *NUDT21* on phenotypes of NSCLC cell and found that knockdown of *NUDT21* promotes NSCLC cell proliferation and migration (Figure S12, Supporting Information). Therefore, we selected NUDT21 as candidate RBP for further investigation.

To experimentally verify that rs9606 influences binding affinity of NUDT21 for *LYRM4*, we performed RNA pull down assays using RNA probes encompassing risk T allele or nonrisk G allele of rs9606. We found NUDT21 was preferentially bound to nonrisk G allele of rs9606 at LYRM4 (LYRM4[G]), which was further validated by RNA immunoprecipitation (RIP) assay (Figure 4E,F). We next examined the regulatory effects of NUDT21 on *LYRM4* APA process through 3′RACE assay. After siRNA‐mediated knockdown of NUDT21, we observed significant 3′UTR shortening of *LYRM4* transcript and the differences in percentage of *LYRM4* short 3′UTR isoform between LYRM4[T] and LYRM4[G] groups were attenuated (Figure 4G,H). Furthermore, the differences in LYRM4 mRNA and protein expression levels between LYRM4[T] and LYRM4[G] were significantly attenuated upon NUDT21 knockdown (Figure 4I,J). In parallel, we measured *LYRM4* mRNA stability, finding that knockdown of NUDT21 stabilized *LYRM4* mRNA and diminished differences of half‐lives between LYRM4[T] and LYRM4[G] groups (Figure 4K). Collectively, these results suggested that NUDT21 preferentially bind to nonrisk G allele of rs9606 to regulate APA process of *LYRM4*, leading to 3′UTR lengthening and decreased LYRM4 expression by destabilized its mRNA.

### 2.9 Risk T allele at *LYRM4* Promotes Malignant Phenotypes of NSCLC Cells through Suppressing Ferroptosis

**Figure 5:**
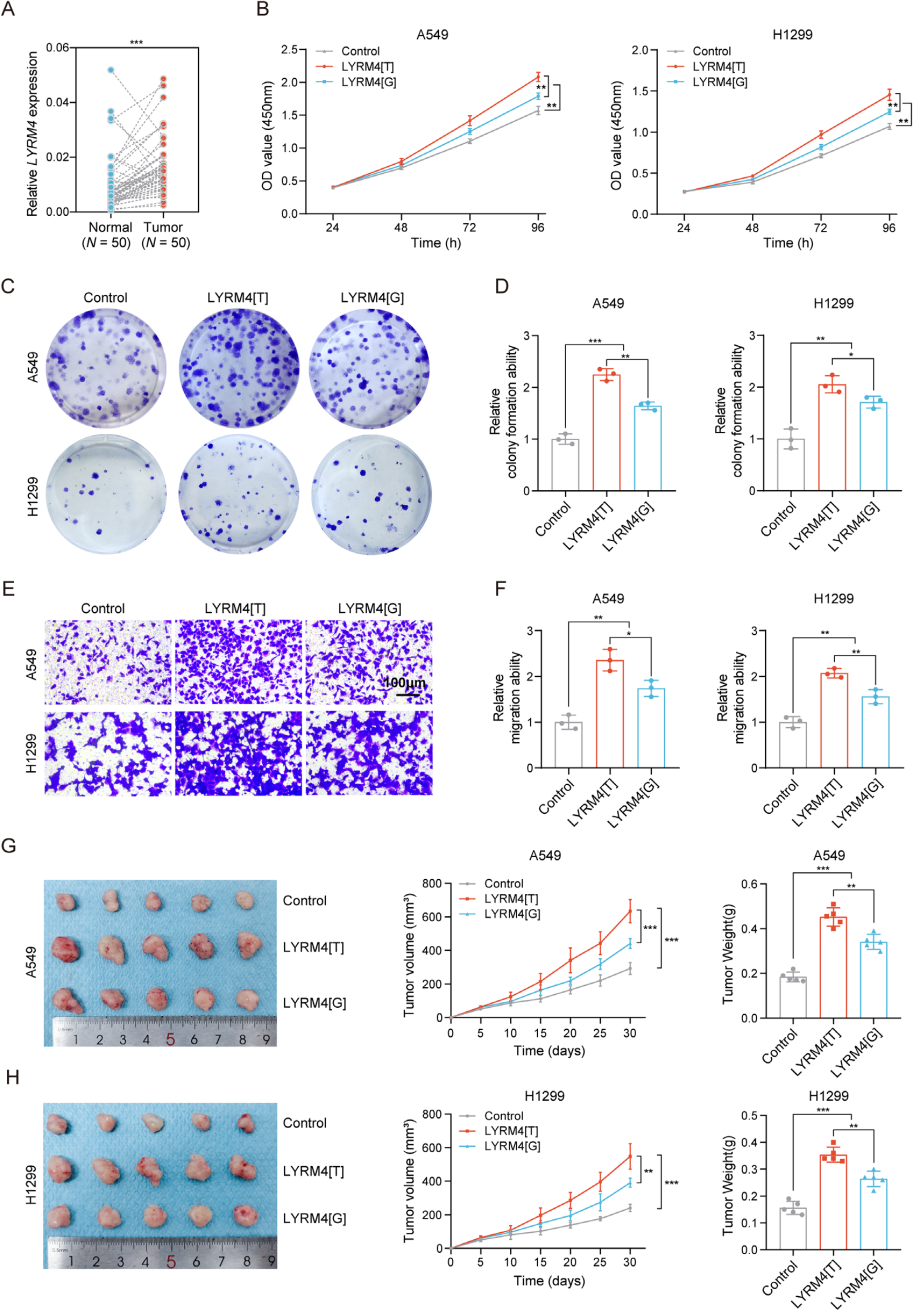
Risk T allele at *LYRM4* drives NSCLC cell proliferation and progression in vitro and in vivo. A) The expression of *LYRM*4 in our own NSCLC tissues and paired adjacent normal tissues. *P‐*value was calculated by Wilcoxon rank‐sum test. B) The effect of LYRM4[T] and LYRM4[G] overexpression on in vitro cell proliferation determined by CCK‐8 assays. Results were shown as the mean ± SD from three independent experiments. C,D) The effect of LYRM4[T] and LYRM4[G] overexpression on colony formation ability of NSCLC cells in vitro. Representative pictures C) and quantification D) of colony formation ability were shown. Results were shown as the mean ± SD from three independent experiments. E,F) The effect of LYRM4[T] and LYRM4[G] overexpression on migration ability of NSCLC cells in vitro. Representative images E) and quantification F) of cell migration ability were shown. Results were shown as the mean ± SD from three independent experiments. G,H) Representative images, growth curves, and tumor weight of xenografts injected with NSCLC cells transfected with LYRM4[G], LYRM4[T], or the control vector. Results were shown as mean ± SD from 5 mice of each group. **P < 0.01 and ***P < 0.001were calculated using Student's *t*‐test.

To further explore biological function of LYRM4 in NSCLC, we first compared the mRNA expression of *LYRM4* in our own NSCLC and adjacent normal tissues, and observed that *LYRM4* was significantly highly expressed in NSCLC tissues (**Figure** **5**A). Then, we overexpressed *LYRM4* with different rs9606 alleles and explore its effect on tumor phenotypes in vitro and in vivo. We found that overexpression of *LYRM4* promoted cell proliferation, migration, and colony‐forming abilities of NSCLC cells (Figure 5B–F), suggesting the oncogenic role of LYRM4 in NSCLC. Notably, compared with LYRM4 containing nonrisk G allele of rs9606 (LYRM4[G]), LYRM4 containing risk T allele of rs9606(LYRM4[T]) showed stronger effects on promoting cell proliferation, migration, and colony‐forming abilities (Figure 5B–F). Consistent with our in vitro findings, we found that LYRM4[T] overexpression facilitated tumor formation in vivo, compared with LYRM4[G] overexpression and control group (Figure 5G,H). Taken together, these in vitro and in vivo results suggest that *LYRM4* may acts as an oncogene in NSCLC development. Overexpression of LYRM4[T] more strongly promotes the malignant phenotypes of NSCLC cancer cells compared to LYRM4[G].

**Figure 6:**
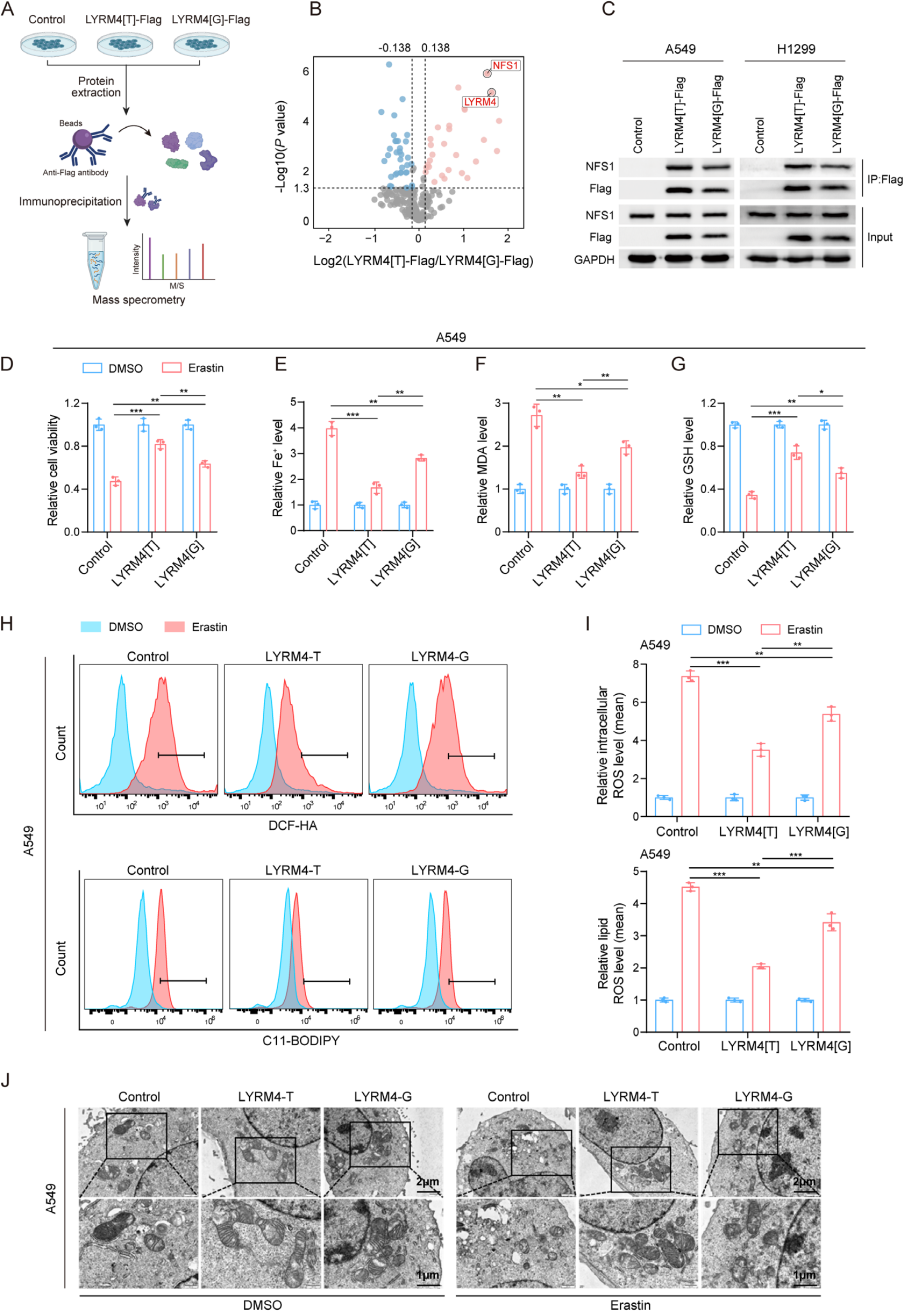
Risk T allele at *LYRM4* suppressing ferroptosis activity to promote malignant phenotypes of NSCLC cells. A) Schematic of LYRM4 interactor identification through proteomic screening by mass spectrometry in A549 cells. This figure was created with BioRender (www.biorender.com). B) Immunoprecipitation followed by proteomic screening showed increased binding of NFS1 in LYRM4[T] overexpression group compared to LYRM4[G] overexpression group. Horizontal line indicates the *P* < 0.05 cutoff. Vertical lines mark the fold change > 1.1 or < 0.9. C) Coimmunoprecipitation followed by western blotting analysis confirmed the interaction between NFS1 and LYRM4 isoforms. D–G) Cell viability D), Fe^2+^ levels E), MDA levels F), and GSH levels G) were measured. A549 cells were transfected LYRM4[T], LYRM4[G], and control vector. After transfection, cells were treated with DMSO or erastin (10 µm) for 24 h. Results were shown as the mean ± SD from three independent experiments. H,I) Intracellular ROS and lipid ROS levels were detected using flow cytometry with DCFH‐DA and BODIPY staining, respectively. A549 cells were transfected with indicated DNA constructs. After transfection, cells were treated with DMSO or erastin (10 µm) for 24 h. Representative images H) and quantitative static results I) were shown. Results were presented as the mean ± SD from three independent experiments. J) Morphological changes of mitochondria were detected by transmission electron microscopy. A549 cells were transfected with indicated DNA constructs, followed by treatment with DMSO or erastin (10 µm) for 24 h. *P* values were calculated using Student's *t*‐test. *, *P* < 0.05; **, *P* < 0.01; ***, *P* < 0.001.

Previous studies have demonstrated that LYRM4 (also known as ISD11) is crucial for the function of iron–sulfur (Fe–S) cluster assembly complex.^[^ ^26^ ^]^ Since Fe–S cluster assembly complex plays a key role in Fe–S cluster biogenesis and ferroptosis, we wondered whether LYRM4 promotes cell proliferation through modulating ferroptosis. To test this hypothesis, we performed immunoprecipitation experiments followed by mass spectrometry (MS) analysis. We identified NFS1 as a potential interactor of LYRM4, and observed an increased binding of NFS1 in LYRM4[T] overexpression group compared to LYRM4[G] overexpression group (**Figure** **6**A,B). Coimmunoprecipitation followed by western blotting analysis further confirmed the direct interaction between LYRM4 and NFS1, and showed LYRM4[T] significantly increased the interaction compared to LYRM4[G] (Figure 6C). As the essential role of NFS1‐LYRM4 complex in the process of Fe–S cluster biogenesis has been reported,^[^ ^27^ ^]^ it is plausible to postulate that LYRM4 is functionally linked to ferroptotic activity. We then overexpressed *LYRM4* with different rs9606 alleles and found that *LYRM4* overexpression significantly inhibited Erastin‐induced ferroptosis of NSCLC cells, which was confirmed by increased cell viability, decreased accumulation of ferroptosis biomarkers Fe^2+^, MDA, cellular ROS, lipid ROS, and increased GSH level (Figure 6D–I; and Figure S13A–F, Supporting Information). Compared with LYRM4[G] overexpression and control group, LYRM4[T] overexpression group was more resistant to Erastin‐induced ferroptosis (Figure 6D–I; and Figure S13A–F, Supporting Information). Morphological change in cells and organelles is another indicator of ferroptosis. Consistently, transmission electron microscopy revealed significant shrunken mitochondria and increased membrane density in NSCLC cells after erastin treatment (Figure 6J; and Figure S13G, Supporting Information). Overexpression of LYRM4[T] partly alleviated these erastin‐induced morphological changes in NSCLC cells, compared with control and LYRM4[G] overexpression group (Figure 6J; and Figure S13G, Supporting Information). In summary, these findings indicated that LYRM4[T] overexpression confers NSCLC cells with stronger resistance to ferroptosis compared to LYRM4[G] and LYRM4 act as an oncogene promoting the malignant phenotypes of NSCLC cells through suppressing ferroptosis.

## 3 Discussion

**Figure 7:**
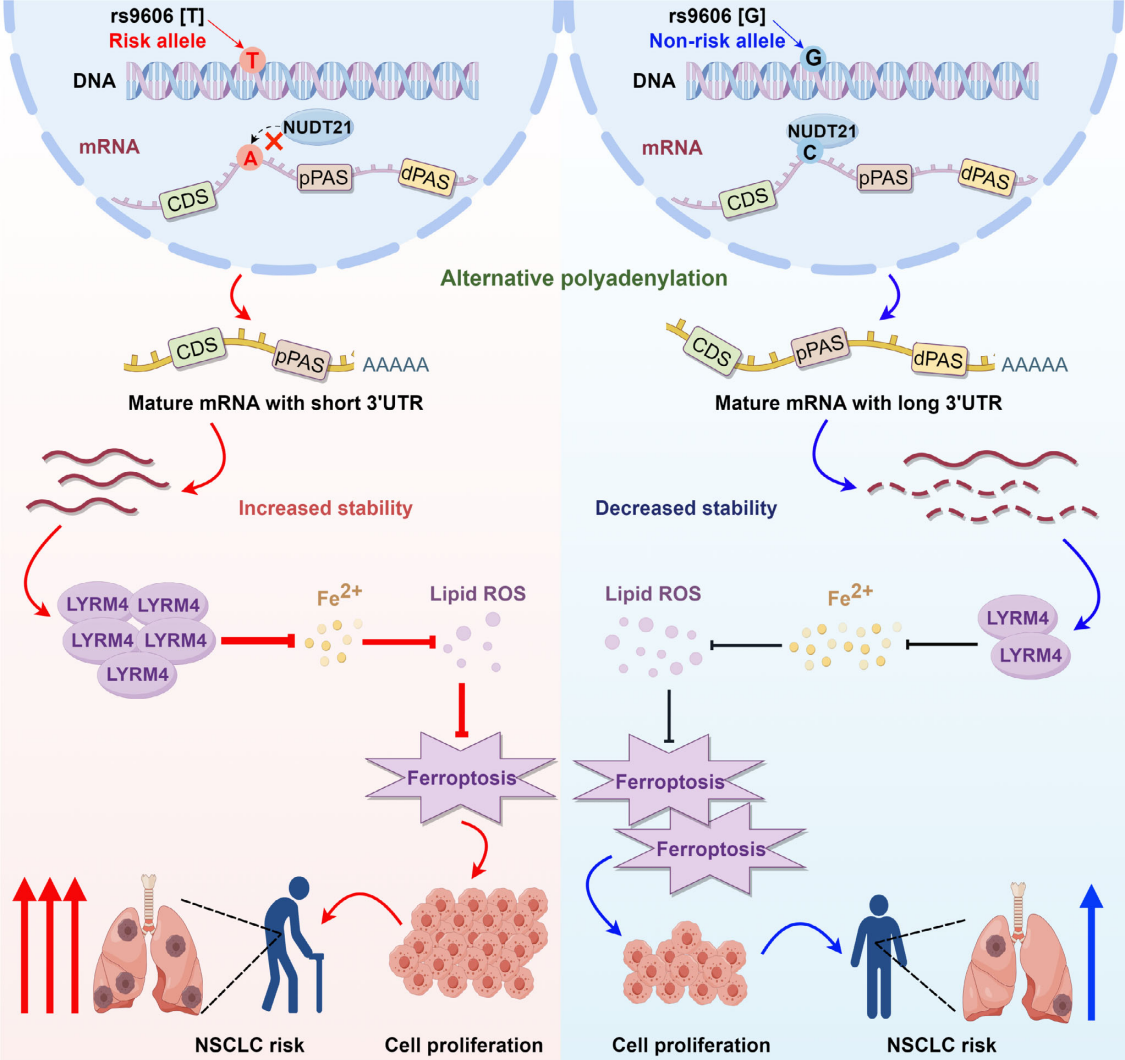
Graphical representation summarizing the mechanism of apaQTL variant rs9606 contributes to NSCLC risk. Compared with the nonrisk G allele of rs9606, the risk T allele of rs9606 decreases the binding affinity of NUDT21 for *LYRM4*, leading to 3′UTR shortening of *LYRM4* mRNA. Shortening of the *LYRM4* 3′UTR leads to increased expression of LYRM4 through stabilization its mRNA. The increased expression of *LYRM4* promoted malignant phenotypes of NSCLC cells through suppressing ferroptosis, which provided functional evidence supporting our population findings that apaQTL variant rs9606[T] allele contribute to increased risk of NSCLC. The figure was created by Figdraw (www.figdraw.com).

In the present study, we performed a large‐scale apaQTL analysis in 1010 NSCLC patients from TCGA. By analyzing genotype and APA events data from TCGA, we identified a total of 117 161 apaQTL SNPs and 2109 corresponding targeted genes in NSCLC. Further integrated analysis of apaQTLs with GWAS suggested the role of APA as an important link from genetic variation to NSCLC risk. Of note, we proved that integration of apaQTL variants with traditional GWAS‐derived PRS improves risk prediction for NSCLC in multiple independent cohorts. Finally, through large‐scale population study and biological experiments, we identified a causal apaQTL variant rs9606 that was significantly associated with NSCLC risk and *LYRM4* APA process. Mechanistically, risk T allele of rs9606 facilitate alternative polyadenylation of *LYRM4* and increase usage of *LYRM4* proximal poly(A) sites mediated by NUDT21, leading to shortening of the *LYRM4* 3′UTR and increased expression of *LYRM4*. The increased expression of *LYRM4* promoted malignant phenotypes of NSCLC cells via ferroptosis inhibition, which could explain why rs9606‐T allele contribute to increased risk of NSCLC. Collectively, this study advances our knowledge of the relationships between genetic variants and alternative polyadenylation, therefore providing new insights into regulatory mechanism underlying NSCLC etiology (**Figure** **7**).

Alternative polyadenylation plays an essential role in many physiological processes and is often dysregulated in cancer.^[^ ^9^, ^28^ ^]^ With the emerging evidence indicate that genetic variants that affect APA events contribute to multiple complex disease risk, there is an urgent need to characterize genetic basis and mechanism of APA regulation. In this study, through integrative apaQTL analysis in NSCLC tissues, we identified a set of apaQTLs in NSCLC. Consistent with previous apaQTL studies,^[^ ^11a^ ^]^ we found that these identified apaQTLs were significantly enriched in 3′UTR region. Interestingly, the identified apaQTLs were enriched in multiple active regulatory elements (epigenetic marks and TF binding sites), implying the potential role in gene regulation.^[^ ^29^ ^]^ We also observed the significant enrichment of apaQTLs within binding sites for various RBPs involved in APA regulation. In particular, we experimentally validated a causal apaQTL variant rs9606 alters the binding site for RBP NUDT21, leading to a shift to an LYRM4 isoform with shorter 3′UTR through APA. These findings are consistent with previous studies suggest genetic regulation of APA events as an important molecular mechanism underlying human diseases.^[^ ^30^ ^]^

Molecular quantitative trait loci (xQTL) analysis provides a powerful strategy to interpret functional effects of GWAS variants. xQTLs identified by previous population‐scale studies, such as expression QTLs(eQTLs),^[^ ^31^ ^]^ splicing QTLs(sQTLs),^[^ ^32^ ^]^ and chromatin QTLs(cQTLs),^[^ ^33^ ^]^ have furthered our understanding of the molecular mechanisms linking genetic variants to cancer susceptibility. In our study, we found that several apaQTLs overlapped with eQTLs. This overlap suggests that these apaQTLs may influence gene expression levels either by modulating the APA process alone or by simultaneously regulating gene transcription, similar to classical 3′UTR eQTL SNPs.^[^ ^34^ ^]^ However, the majority of apaQTLs and eQTLs appear to be largely independent. We speculate that eQTL‐independent apaQTLs regulate translation or the cellular localization of target genes without affecting the regulation of gene expression.^[^ ^35^ ^]^ Consistently, our integrated analysis of apaQTLs and NSCLC GWAS data also revealed the important role of apaQTLs in NSCLC risk. Colocalization analyses showed significant enrichment of apaQTLs among NSCLC GWAS loci. Further analysis indicated that apaQTLs can explain some of the missing heritability in NSCLC and are largely distinct from eQTLs. Furthermore, our investigations revealed significant association between an apaQTL variant rs9606 and NSCLC risk, which was further validated in multiple independent cohorts. Mechanistically, rs9606 affects the choice of poly(A) site by NUDT21, resulting in altered mRNA stability of *LYRM4* which ultimately contributes to NSCLC cell proliferation. Thus, large‐scale apaQTL studies in NSCLC and other tissues can discover more functional genetic variants to advance our understanding of genetic regulation of APA and facilitate the translation of GWAS findings to mechanistic understanding.

PRS are playing increasing important roles in research studies to identify individuals at high risk of diseases, such as cardiovascular disease^[^ ^36^ ^]^ and lung cancer.^[^ ^37^ ^]^ However, due to the modest predictive ability for diseases, the clinical application of PRS in disease risk assessment remains limited. In this study, we constructed three PRS models and evaluated the its predictive performance of NSCLC risk. Of note, integration of apaQTL variants with traditional GWAS‐derived PRS improves NSCLC risk stratification across multiple independent cohorts. This finding is consistent with recent studies, which suggested that incorporating functional variants discovered in molecular quantitative trait loci studies improve risk prediction ability of diseases.^[^ ^38^ ^]^ Remarkably, the combined PRS not only optimized the risk stratification for NSCLC in case‐control sets, but also outperformed in separating populations into high risk and low risk of NSCLC respectively in a perspective cohort set. These findings provided additional evidence that apaQTL variants could be used for NSCLC risk assessment, implying the its potential translational utility for NSCLC screening and individualized prevention.

Identifying and annotation of target genes from genetic data will enable mechanistic understanding of disease and development of innovative therapies.^[^ ^39^ ^]^ In this study, we found that target genes affected by apaQTLs were enriched in processes related to carcinogenesis, including oncogenic pathways, immune infiltration, and drug responses. Among these targeted genes, we experimentally validated that rs9606 target gene *LYRM4* directly impacted malignant phenotypes of NSCLC cells in vitro and in vivo. *LYRM4* (also known as ISD11) has an essential role in both iron–sulfur (Fe–S) cluster biogenesis and maintenance of cellular iron homeostasis.^[^ ^27^, ^40^ ^]^ Depletion of *LYRM4* causes deficiency in Fe–S cluster biogenesis, which leads to ferroptotic cell death in cancer cells.^[^ ^41^ ^]^ Our study demonstrated that overexpression of *LYRM4* promotes malignant phenotype of NSCLC cells through regulating ferroptotic activity, which is consistent with previous reports that overexpression of *LYRM4* protects lung cancer cells from undergoing ferroptosis.^[^ ^42^ ^]^ Interestingly, ferroptosis plays critical role in tumor metastasis and immune response. Induction of ferroptosis not only suppresses tumor progression but also augments immunotherapy responses.^[^ ^43^ ^]^ However, our understanding of the interaction between LYRM4‐mediated ferroptosis regulation and tumor immunity remains limited. Therefore, future studies should be warranted to investigate potential role of *LYRM4* in metastasis, tumor microenvironment, and immune response. It will also be interesting to investigate the intriguing potential of the association between SNPs and APA events of noncoding RNAs in the future.

This study has several limitations. First, similar to eQTLs and sQTLs,^[^ ^44^ ^]^ apaQTLs may also be cell type‐specific. Our current study only used bulk RNA‐seq data for analysis, potentially overlooking genetic effect that are cell type‐specific and in turn limiting their functional interpretation. Therefore, further investigations should combine GWAS with single‐cell genomic atlases to provide a more comprehensive understanding of genetic landscape and regulatory mechanism underlie NSCLC. Second, we have identified numerous genetic variants associated with alternative polyadenylation and NSCLC risk, but the functional consequences of these variants are mostly unknown. More functional experiments are required to elucidate the molecular mechanisms underlie genetic control of APA and the following cascades of events that lead to NSCLC. For instance, RNA sequencing (RNA‐Seq) is planned to provide a more comprehensive view of the global impact of rs9606 on gene expression, including noncoding RNAs and other regulatory pathways. Third, apaQTLs identified in this study only explain a small fraction of NSCLC GWAS loci, probably due to genetic regulatory mechanisms beyond alternative polyadenylation. Thus, investigation combined effects of genetic variant associated with other molecular phenotypes (e.g., alternative splicing, N^6^‐methyladenosine, and histone modification) with apaQTL variants is essential to unveil molecular basis of polygenic diseases and traits. Fourth, subsequent research could aim to develop transcriptome‐wide association studies (TWAS) based on QTL results to highlight the role of APA in nominating susceptibility genes. Finally, various environmental and lifestyle factors, including diet, obesity, and physical activity, have been shown to be associated with the NSCLC risk.^[^ ^45^ ^]^ These factors should be adjusted to enhance the accuracy of risk assessment.

## 4 Conclusion

In summary, as a comprehensive apaQTL study in NSCLC thus far, our recent work provides a catalog of apaQTL variants that will be a valuable resource for future studies. We further characterized functional properties of these apaQTL variants, demonstrating its distinct role in NSCLC risk. Functional annotation of apaQTLs and enrichment of their target genes in cancer‐related pathways will inform the interpretation of NSCLC GWAS loci, providing valuable functional hints for potential leading molecular pathways. In particular, we confidently identified an apaQTL variant rs9606 as a causal variant for NSCLC. rs9606 promoted aberrant alternative polyadenylation process of *LYRM4*, which lead to increased expression of oncogene *LYRM4* and thus contribute to NSCLC risk. These findings may help advance our understanding of the molecular mechanisms linking genetic variation to NSCLC susceptibility and will ultimately improve precise prevention and treatment strategies of NSCLC.

## 5 Experimental Section

### Integrative apaQTL Analyses in NSCLC

To quantify dynamic APA events in NSCLC, a well‐established DaPars was utilized for identification APA event indicated by Percentage of Distal polyA site Usage Index (PDUI) for each transcript. The PDUI data measure derived from DaPars were mined from TC3A (http://tc3a.org/). To generate a reliable set of APA events, APA events were filtered according to the following criteria: i) PDUI values with a missing rate ≥ 0.1; ii) PDUI values with a standard deviation ≤ 10%. Ultimately, an average of 4243 APA events in NSCLC were included for further analysis.

Genotype data of NSCLC samples detected by the Affymetrix SNP 6.0 Array were obtained from TCGA portal (https://portal.gdc.cancer.gov/). To fill the missing genotypes of these NSCLC samples, prephase and imputation was conducted by IMPUTE2 (version 2.3.2),^[^ ^46^ ^]^ with 1000 Genomes Project Phase III database as the reference. After imputation, further variants were excluded with imputation confidence score < 0.4, minor allele frequency (MAF) < 5%, SNP missing rate ≥ 5% for best‐guessed genotypes at posterior probability ≥ 0.9, or departure from Hardy–Weinberg Equilibrium (*P* < 1×10^−6^). After imputation and quality control, 4 022 558 SNPs per cancer type were remained for downstream analyses.

After PDUI values and genotype data were obtained, an integrative apaQTL analysis was performed to identify apaQTL SNPs in NSCLC. To account for potential known and other hidden covariates in apaQTL analyses, first principal component analysis (PCA) was performed by using PLINK to correct sample genotype for population structure. Further PEER method was used to adjust for the first 15 PEER factors of PDUI values in each tissue. Other common confounders include age, sex, and tumor stage were also included as known covariates for apaQTL analysis. Then a linear regression framework in FastQTL^[^ ^47^ ^]^ was applied to test associations between SNPs and APA events within a distance of 1 Mb around a candidate SNP as previously described.^[^ ^11b^ ^]^ apaQTL SNPs were subsequently identified with an FDR below 0.05.

### Functional Annotation Comparisons Between apaQTLs and Non‐apaQTLs

To generate the control set of non‐apaQTL SNPs, first SNPs were extracted which were matched to apaQTL SNPs in terms of MAF, LD, and variant type by using a web tool vSampler (http://mulinlab.org/vsampler/). Then, non‐apaQTL SNPs were selected which are not associated with APA events.^[^ ^48^ ^]^ Next, apaQTL and non‐apaQTL SNPs were functionally annotated using a SNP annotation tool SnpEff.^[^ ^49^ ^]^ According to the functional annotation defined by SnpEff, SNPs were classified into following categories: 3′‐untranslated region (UTR), 5′UTR, downstream, upstream, intronic, intergenic, and exonic. The difference in enrichment of apaQTLs and non‐apaQTLs among each functional category was evaluated by the two‐tailed Fisher's exact test with 2×2 table (columns; apaQTL and non‐apaQTL SNPs, rows; SNPs “assigned” and “not assigned” to the particular functional class).

### Functional Enrichment of apaQTLs within Poly(A) Motifs, Functional Elements, and RBP Binding Sites

Putative poly(A) sites of genes undergoing APA were obtained from PolyA^[^ ^50^ ^]^ and UCSC databases. Subsequently, NSCLC apaQTLs which located within the 3′UTR of the associated APA genes were intersected with 50 bp upstream of each poly(A) site. Sequence located within 6 bp upstream or downstream of poly(A)‐overlapping apaQTLs were extracted to identify poly(A) motif‐altering apaQTLs. Enrichment analyses of apaQTL SNPs among poly(A) motifs were conducted by two‐tailed Fisher's exact test with the 2×2 table (columns; apaQTL and non‐apaQTL SNPs, rows; SNPs within and not within poly(A) motifs).

To assess whether the identified apaQTL SNPs were enriched among functional regulatory elements in the genome, functional enrichment analysis of apaQTL SNPs was performed. In brief, annotation files for epigenetic histone marks of promoters and enhancers, and transcription factor‐binding sites (TFBs) were obtained from ENCODE.^[^ ^16b^ ^]^ Then, overlap of apaQTL SNPs with each regulatory element peaks using BEDTools was explored.^[^ ^51^ ^]^ For enrichment analysis, two‐tailed Fisher's exact test with 2×2 table (columns; apaQTL and non‐apaQTL SNPs, rows; SNPs within and not within regulatory element) was used.

To predict potential APA regulators that function through binding to apaQTLs, enrichment analysis of apaQTLs among RBP binding sites was performed. Enhanced crosslinking and immunoprecipitation followed by sequencing (eCLIP‐seq) data were downloaded from ENCODE data portal (https://www.encodeproject.org). Then, whether each apaQTL SNP falls within RBP binding peaks using BEDtools was tested. The statistical significance of the enrichment analysis was evaluated using two‐tailed Fisher's exact test with 2×2 table (columns; apaQTL and non‐apaQTL SNPs, rows; SNPs within and not within the RBP binding site).

### Enrichment of apaQTLs within NSCLC GWAS loci

To test for enrichment of apaQTL SNPs among NSCLC GWAS loci, GWAS SNPs were downloaded associated with NSCLC risk from the GWAS catalog (https://www.ebi.ac.uk/gwas/). SNPs with genome‐wide significant association *P* < 5×10^−8^ were included in the analyses (Table S7, Supporting Information). SNPs that are in LD with an index SNP at *r* ^2^ ≥ 0.2 were defined as an associated genomic locus. Subsequently, the BEDtools were used to test whether an SNP was located within NSCLC GWAS loci. The enrichment of apaQTL SNPs among NSCLC loci was evaluated using two‐tailed Fisher's exact test with 2×2 table (columns; apaQTL and non‐apaQTL SNPs, rows; SNPs within and not within the GWAS loci).

### Partitioned Heritability Analysis

To estimate the heritability of apaQTLs for NSCLC, GWAS results were first downloaded from the OncoArray Consortium lung cancer studies (dbGaP accession number: phs001273.v3). Next, GWAS SNPs were extracted that also belong to apaQTLs and plotted the quantile–quantile plot of the GWAS *P*‐values for those SNPs. Similarly, GWAS SNPs were plotted that also belong to eQTLs for comparison. Genome wide *P*‐values were plotted as a control. The heritability enrichment of apaQTLs was estimated by stratified LD Score Regression using (S‐LDSC, v1.0.1, https://github.com/bulik/ldsc). The enrichments were calculated as the proportion of heritability over the proportion of SNPs, and then the standard errors were estimated and used for *P*‐value calculation.^[^ ^52^ ^]^

### Characterization of aGenes

To explore the potential role of genes affected by apaQTLs (aGenes) in tumorigenesis, further these aGenes in terms of biological function, somatic copy number alterations (SCNAs), and aGenes associated immune cell landscape and drug response were characterized. To examined functional enrichment of aGenes in biological pathways, Kyoto Encyclopedia of Genes and Genomes (KEGG) analysis was applied.^[^ ^53^ ^]^ *P* < 0.05 was set as the threshold to identify significantly enriched pathways. Somatic copy‐number alteration (SCNA) data for NSCLC analyzed by GISTC2 were downloaded from the TCGA data portal, to reveal relevant genetic alterations of aGenes. Immune cell infiltration data of NSCLC was obtained from Tumor Immune Estimation Resource (TIMER2, http://timer.cistrome.org/). The associations between immune infiltrates and aGene expression were evaluated by Spearman's rank correlation analysis. To explore associations between expression of aGenes and drug response, gene expression, and drug IC50 data were downloaded from the Genomics of Drug Sensitivity in Cancer (GDSC, released June 2020, https://www.cancerrxgene.org/). Then, the associations were evaluated by Spearman's rank correlation analysis and FDR < 0.05 were defined as significant.

### Study Subjects in Association Analysis between apaQTLs and NSCLC Risk

To screen for potential functional apaQTL variants that associated with NSCLC risk, a multicenter study in multiple cohorts was performed. In the discovery stage, a GWAS array analysis using data from the OncoArray Consortium lung cancer studies (dbGaP accession number: phs001273.v3.p2) was conducted. After excluding 1815 small cell lung cancer samples, a total of 16 665 NSCLC cases and 14 101 healthy controls were remained. Furthermore, 1 eligible control from subjects without invasive NSCLC was selected by nearest neighbor matching in R package MatchIt, with enrollment gender and age group as matching criteria. Finally, 13 835 NSCLC cases and 13 835 matched controls were included for further analysis. The demographic characteristics of samples were listed at Table S1 (Supporting Information).

In the replication stage, genotyping analysis of the own NSCLC samples was performed by TaqMan Assays and ABI Prism 7900HT system. Quality control was implemented as described in previous study.^[^ ^54^ ^]^ Three independent population cohorts from China were used for evaluating the association between candidate apaQTL variant rs9606 and NSCLC risk. In the cohort I, a total of 1700 cases and 1700 controls were recruited from the First Affiliated Hospital of Zhengzhou University (Zhengzhou, China). In the cohort II, a total of 2809 cases and 2809 controls were recruited from Tongji Hospital of Huazhong University of Science and Technology (HUST, Wuhan, China). In cohort III, a total of 2500 cases and 2500 controls were recruited from Peking University Cancer Hospital and Institute. The details of demographic characteristics of all samples have been described in Table S4 (Supporting Information). This study was approved by the Institutional Review Board of Tongji hospital (TJ‐IRB20230707), and each participant provided written informed consent at recruitment.

In addition, genotype data from the UK Biobank (UKB) and Prostate, Lung, Colorectal, and Ovarian Cancer Screening Trial (PLCO) datasets were adopted to strengthen the finding in the own samples. In UKB dataset, NSCLC cases were defined as subjects with primary invasive NSCLC diagnosed or NSCLC deaths according to ICD‐10 (C33, C34, and C39.9) codes. For each case, 4 eligible controls were selected from subjects without invasive NSCLC by nearest neighbor matching in R package MatchIt, with enrollment age, race/ethnicity, and sex as matching criteria. Finally, 3622 NSCLC cases and 14 488 matched controls were included (Table S2, Supporting Information). In PLCO dataset (accession number: phs000346.v2. p2, phs001554.v1. p1, phs001286.v2. p2, and phs001524.v1. p1), 1712 NSCLC cases and 6848 healthy controls were included to evaluate the association between rs9606 and NSCLC risk. Demographic characteristics of all subjects were displayed in Table S3 (Supporting Information).

### Generation of Polygenetic Risk Scores to Predict NSCLC Risk

For the PRS development, the summary statistics was included from an inverse variance‐weighted fixed‐effects meta‐analysis performed with METAL based on the GWAS from EAS (Biobank Japan NSCLC GWAS; 4444 NSCLC cases and 174282 healthy controls) and EUR populations (FinnGen NSCLC GWAS; 5842 NSCLC cases and 287137 controls). The study employed three distinct PRS models using the selected sets of variants as follows: 1) Standard PRS: This model incorporated 120 variants previously identified in numerous studies to be significantly associated with NSCLC risk at the genome‐wide level. 2) apaQTL‐associated PRS: This model focused on 366 independent NSCLC apaQTL variants with MAF>0.05 by clumping from 117 161 apaQTL variants. 3) Combined PRS: This model integrated all 486 independent variants from both standard PRS and apaQTL‐associated PRS. To ensure the efficiency of PRS models, LD‐clumping procedure in PLINK v.1.90 was performed to remove highly correlated variants. The detail clumping parameters of PRS were as follow: –clump‐*p* 5×10^−8^, –clump‐*r* ^2^ 0.10 –clump‐kb 250. In brief, summary statistics (effect alleles, ORs, and *P* values for each variant) from the NSCLC GWAS meta‐analyses were coordinated with the genotype data of case‐control population or cohort population. Then the PRS was constructed by utilizing clumping and thresholding (C+T) method, which is the most commonly used approach for PRS calculation. Individuals were additively scored in a weighted pattern based on the number of risk alleles they carry for each variant in the PRS.

Subsequently, predictive performance of three PRS models described above in large‐scale case‐control sets and a perspective cohort set was assessed. In brief, summary statistics (effect alleles, ORs, and *p* values for each variant) was used from all GWASs, including UK biobank, OncoArray Consortium lung cancer studies, and PLCO. The association between PRSs and NSCLC risk among case‐control population were then calculated using logistic regression analysis with low‐PRS (bottom 20%) as a reference, adjusting for age and gender. Subjects were classified into 5 groups according to the distribution of PRS, the dose‐response relationship (*P*_trend_) and risk of NSCLC (ORs) for each group were calculated using logistic regression. For the perspective UK biobank cohort study, Cox proportional hazards regression model was used to obtain hazard ratios (HRs) and 95% CI by comparing the incident risk of participants who had a high (top 33%) genetic risk with the incident risk of those who had a low (bottom 33%) genetic risk.

### Datasets for PRS Application

Predictive performance of PRS models was assessed for NSCLC risk stratification among large‐scale case‐control sets and cohort set. For case‐control sets, it was included from UK biobank dataset (3622 NSCLC cases and 14488 matched controls), OncoArray Consortium lung cancer studies (13835 NSCLC cases and 13835 healthy controls), and PLCO studies (1712 NSCLC cases and 6848 matched healthy controls). In terms of cohort set, the performance of these PRSs for NSCLC incidence in UK Biobank dataset was tested. UK biobank is a large‐scale database and cohort containing genetic and health information with follow‐up from more than 500 000 UK participants between April 2006 and December 2010. Participants with missing genotype data or covariates (sex, age) at baseline or a history of other cancers according to the first cancer diagnosis were excluded from the dataset. The follow‐up time was calculated from baseline assessment to the first diagnosis of NSCLC, loss to follow‐up, and death or last follow‐up (October 1, 2022). Finally, 3777 incident NSCLC cases and 415 212 cancer‐free normal individuals during follow‐up were included for further evaluation.

### Cell Culture

The human NSCLC cell lines A549 and H1299 were obtained from the Cell Bank of Chinese Academy of Sciences (Shanghai, China). Cells were cultured in RPMI 1640 (HyClone, USA) containing 10% fetal bovine serum (Gibco, USA) and 1% penicillin–streptomycin (HyClone, USA) and incubated at 37 °C with 5% CO_2_ and frozen in commercial cell freezing solution (Abkkine, China) at ‐80 °C. All cell lines used were authenticated by short tandem repeat (STR) profiling (Applied Biosystems, USA), and mycoplasma contamination was not detected (MycoAlert, USA).

### Plasmids, siRNAs, and Lentiviruses

The plasmids expressing full‐length *LYRM4* cDNA containing the rs9606[T] (LYRM4[T]) or rs9606[G] allele (LYRM4[G]) and the plasmids expressing *LYRM4* cDNA with short 3′UTR or long 3′UTR were constructed using pcDNA3.1(+) vector by Tsingke (Beijing, China). The *NUDT21*‐targeting siRNA and nontargeting siRNA control were commercially synthesized by AuGCT Biotech Co., Ltd. (Beijing, China). The siRNA sequences are listed in Table S4 (Supporting Information). For plasmid transfection and cotransfection with siRNA, Lipofectamine 3000 (Invitrogen, USA) was used as per manufacturer's instructions.

Lentivirus for full‐length *LYRM4* cDNA containing the rs9606[T] (LYRM4[T]) or rs9606[G] allele (LYRM4[G]) were purchased from OBIO technology and infected with A549 and H1299 cells for in vivo experiments.

### 3′‐Rapid Amplification of cDNA Ends (3′RACE) Assay

The 3′RACE assay was performed with the HiScript‐TS 5′/3′ RACE Kit (Cat#: RA101‐01, Vazyme) according to the manufacturer's instruction. In brief, after transfecting with LYRM4[T], LYRM4[G], and empty vector plasmids into A549 and H1299 cells for 48 h, total RNA was extracted using TRIzol reagent (Invitrogen, USA) and reverse transcribed to first‐strand complementary DNA. The gene‐specific primer (GSP) of *LYRM4* and universal primer mix (UPM) were used to perform the first‐round PCR, and nested GSP of *LYRM4* and Nested Primer were used to perform the nested PCR. The amplified products of 3′RACE were separated with 1% agarose gels, and then the target bands were excised and recovered using FastPure Gel DNA Extraction Mini Kit (Cat#: DC301‐01, Vazyme). Next, the purified products were cloned into pCE2 TA/Blunt‐Zero Vector using 5 min TA/Blunt‐Zero Cloning Kit (Cat#: C601‐01, Vazyme) and identified by Sanger sequencing. The UPM and Nested Primer were provided by the HiScript‐TS 5′/3′ RACE Kit. The sequences of GSP and nested GSP designed for *LYRM4* were listed in Table S4 (Supporting Information).

### Quantitative Reverse Transcription PCR (qRT‐PCR)

Total RNA was isolated from NSCLC cell lines, NSCLC tissues, or adjacent normal tissues using TRIzol reagent (Invitrogen, USA) and reverse transcribed to first‐strand complementary DNA using the HiScript III RT SuperMix (Vazyme, China). Quantitative real‐time PCR (qPCR) was conducted with SYBR Green qPCR Master Mix (Selleck, USA). The mRNA expression was normalized to *GAPDH*. The primer sequences used in qPCR were listed in Table S5 (Supporting Information).

### Western Blotting

NSCLC cells were lysed using RIPA buffer with a mixture of protease inhibitors PMSF (Beyotime, China) and cocktail (MedChem Express, USA). The lysates were sonicated and centrifuged, supernatant was quantified using a BCA assay kit (Beyotime, China). The proteins were separated by SDS‐PAGE and transferred to PVDF membrane, then incubated with antibodies against LYRM4 (Cat# PA5‐56448, Invitrogen), NUDT21 (Cat# 10322‐1‐AP, Proteintech), GAPDH (Cat# 10494‐1‐AP, Proteintech), β‐actin (Cat# 20536‐1‐AP, Proteintech) and following incubated with antimouse or antirabbit secondary antibodies, then detected with ECL reagent (NCM Biotech, China).

### RNA Stability Assay

Cells were seeded and transfected with different plasmids, in 6‐well plates. After 36 h of transfection, cells were treated with actinomycin D (5 µg mL^−1^, Cat# S8964, Selleck) and the total RNA was exacted at indicated time points. The half‐life time of mRNA was estimated as previously described.^[^ ^55^ ^]^ The decay curves of mRNA were plotted using the nonlinear fitting with the equation: *N*_t_/*N*_0_ = e^−^ *^k^* ^t^. The transcription inhibition time is t, the degradation rate is *k*, and the quantities of RNA at time t and time 0 are *N*_t_ and *N*_0_. The mRNA half‐life was determined using the following equation: T_1/2_ = ln 2/*K*.

### RNA Pull‐Down Assay

Biotinylated RNA probes spanning the rs9606 site of 50 bp were designed and commercially synthesized. RNA pull‐down experiments were performed using RNA pull‐down Kit (BersinBio, cat. no. Bes5102, China). According to the manufacturer's instructions, the cellular protein extracts from at least 1×10^7^ NSCLC cells were incubated with 50 pmol biotinylated RNA probes and 50 µL streptavidin magnetic beads with rotation. After incubation period, the beads were washed with wash buffer and captured with magnetic rack. The retained proteins in the beads were eluted with biotin elution buffer, followed by mass spectrometry identification and western blot analysis.

### Liquid Chromatography Tandem Mass Spectrometry (LC‐MS) Analysis

The bead samples from RNA pull‐down or immunoprecipitation were collected and washed with cold PBS, followed by digestion with sequencing‐grade trypsin (Promega). Afterward, the MS data were collected using a LC‐MS system that combines the Q Exactive HF mass spectrometer with the UltiMate 3000 RSLCnano liquid phase. MS data were analyzed using MaxQuant software (V1.6.6) with the Andromeda database search algorithm, and the database for search was the UniProt human proteome reference database.

### Human NSCLC Samples Collection

A total of 50 NSCLC and adjacent normal lung tissues from patients who underwent scheduled surgery in Wuhan Tongji hospital were obtained. Patients who donated samples did not receive chemotherapy, radiation therapy, targeted therapy, or immunology therapy before surgical resection. The clinical information of all samples was provided in Table S6 (Supporting Information). The sample collection was conducted in accordance with the ethical principles in Declaration of Helsinki, and approved by the Ethics Committee of Tongji Medical College, Huazhong University of Science and Technology.

### RNA Immunoprecipitation (RIP) Assay

For RIP assays, A549 and H1299 cells were transfected with LYRM4[T] or LYRM4[G] plasmids. Afterward, cells were lysed with precold RIP lysis buffer on ice. The lysates were centrifuged, part of the supernatant was served as the input control, the remaining supernatant was incubated with anti‐NUDT21 (Cat# 10322‐1‐AP, Proteintech) or isotype control (IgG) and protein A/G beads and rotated at 4 °C overnight. Next, the beads were washed with precold RIP wash buffer and collected using magnetic rack, and RNA was extracted from the beads and input using Trizol. The RNA abundance of *LYRM4* mRNA was further analyzed through qRT‐PCR method and normalized to input.

### Cell Counting Kit‐8 (CCK‐8) Assay

Cell proliferation and viability were measured by CCK‐8 kit. The pretransfected cells were seeded in 96‐well plates, with each well containing 100 µL medium of 3000 cells. After cultured for 24, 48, 72, and 96 h, cell proliferation were determined using CCK8 reagent (MedChem Express, USA) by measuring the absorbance at 450 nm wavelength with a microplate reader.

### Colony Formation Assay

For the colony formation assay, the pretransfected cells (200 cells per well) were seeded in 6‐well plates. Following a period of 10–14 days, the cell colonies were fixed with 4% paraformaldehyde and stained with 0.1% crystal violet. After washing with water, the visible colonies were imaged and counted by ImageJ as previously described.^[^ ^56^ ^]^

### Migration Assay

The migration assay was performed by employing Transwell plates (Corning, USA; 8 µm pore size). A total of 0.2 mL (2 × 10^4^ cells) of cell suspension with serum‐free medium was added into the upper chambers of Transwell insert, and 0.6 mL medium containing 10% FBS was placed to the lower chamber. After 24 h of incubation, migrated cells were fixed with 4% paraformaldehyde, and subsequently stained with 0.1% crystal violet.

### Dual‐Luciferase Reporter Assay

The sequences of *LYRM4* short 3′UTR and long 3′UTR were synthesized and subcloned into pmirGLO reporter vector (Promega, USA). To generate vector that expression of only long 3′UTR of *LYRM4*, the proximal PAS sequence was mutated. The sequences of *LYRM4* 3'UTR containing the rs9606 T allele or G allele were cloned into pmirGLO reporter vector. A549 and H1299 cells were transfected with above reporter plasmid. 48 h after transfection, the renilla and firefly luciferase activities were measured with a Dual‐Luciferase Reporter Assay Kit (Cat# DL101‐01, Vazyme) according to the manufacturer's protocol. The activity values of firefly luciferase were normalized against that of Renilla luciferase.

### Coimmunoprecipitation (CO‐IP) Assay

Cells transfected with indicated plasmids in 10 cm plate were washed with precold PBS and lysed with IP lysis buffer, and then the supernatant was collected after centrifugation. Cell supernatant was incubated with anti‐FLAG (Cat# F1804, Sigma‐Aldrich) and protein A/G magnetic beads overnight at 4 °C with rotation. After washing the magnetic beads with IP wash buffer, the beads were prepared for mass spectrometry or western blot analysis.

### Cellular Iron Detection

Intracellular ferrous iron (Fe^2+^) levels were detected with the Cell Ferrous Iron Colorimetric Assay Kit (E‐BC‐K881‐M, Elabscience, China) and the absorbance at 593 nm was calculated as the intracellular Fe^2+^ levels. The iron concentration was determined using a standard calibration curve based on the measured absorbance at 593 nm.

### Malondialdehyde (MDA) and Glutathione (GSH) Detection

The relative intracellular MDA and GSH levels were detected using the commercial MDA assay kit (BC0020, Solarbio, China) and GSH assay kit (BC1170, Solarbio, China) respectively, according to the manufacturer's protocol. The MDA and GSH contents were calculated by measuring the absorbance at 532 and 412 nm, respectively.

### Intracellular ROS and Lipid Peroxidation Detection

The lipid ROS level was used to evaluate lipid peroxidation. Intracellular ROS levels and lipid ROS levels were detected using DCFH–DA (Beyotime, China) and C11 BODIPY 581/591(Invitrogen, USA) respectively. Briefly, after treatment, cells were incubated with DCFH‐DA (10 µM) or C11‐BODIPY (10 µm) for 30 min at 37 °C in the dark. After thrice washing with serum‐free medium, the cells were observed and photographed using a fluorescence microscopy, or analyzed by flow cytometry to measure the fluorescence intensity.

### Transmission Electron Microscopy (TEM)

After the indicated treatment, cells were fixed with 2.5% glutaraldehyde at 4 °C overnight. Then, the samples were dehydrated, embedded in epoxy resin and cut into ultrathin sections. After staining with uranyl acetate and lead citrate, the morphology of mitochondria was observed by TEM (JEOL, Japan).

### Animal Studies

Five weeks old male BALB/c nude mice were purchased from Nanjing Gempharmatech Co., Ltd, China and raised in specific pathogen‐free (SPF) conditions. A549 or H1299 cells (5 ×10^6^ in 100 µL PBS) with stable overexpression of *LYRM4*[T] and *LYRM4*[G] were subcutaneously injected into the armpit of each mouse. Tumor size was measured using a caliper and the tumor volume was calculated according to the formula: volume = (length × width^2^)/2. All animal experiments were approved by the Animal Ethics Committee of Tongji Hospital (202 311 040).

### Statistical Analysis

Statistical comparisons were assessed using Student's *t*‐test or *P*earson's *χ* ^2^ test for continuous and categorical variables, respectively. For candidate variant association analysis, unconditional multivariable logistic regression was performed to estimate odds ratios (ORs) and 95% confidence intervals (CIs), with adjustment for gender, age, smoking status, and drinking status. Multiple genetic models that include allelic, dominant, recessive, and additive genetic models were used to assess the significance associations between rs9606 genotypes and NSCLC risk. Other methods used for calculating utility and effectiveness of PRS can be found in relevant method part. Statistical analyses for gene expression in NSCLC and adjacent normal tissues were calculated using Wilcoxon rank‐sum test. Detailed statistical methods used in each functional experiment were illustrated in the relevant figure legends. Asterisks in the figures define the significance level (*P < 0.05; **P < 0.01; and ***P < 0.001). All statistical analyses were performed using SPSS software (21.0) or R (3.30).

## Conflict of Interest

The authors declare no conflict of interest.

## Supporting information



Supporting Information

Supplemental Table 1

## Data Availability

The data that support the findings of this study are available in the supplementary material of this article.
