# Supplementary material for: Genetic Regulation of Alternative Polyadenylation Provides Novel Insights into Molecular Mechanisms Underlying Non‐small Cell Lung Cancer
Source: Adv Sci (Weinh). 2025 Apr 26;12(26):2502008. doi: 10.1002/advs.202502008 (PMC12245006; doi:10.1002/advs.202502008)
Supplement: Supplementary file 1 — Supporting Information [file ADVS-12-2502008-s001.docx]

**Supplementary Figures:**


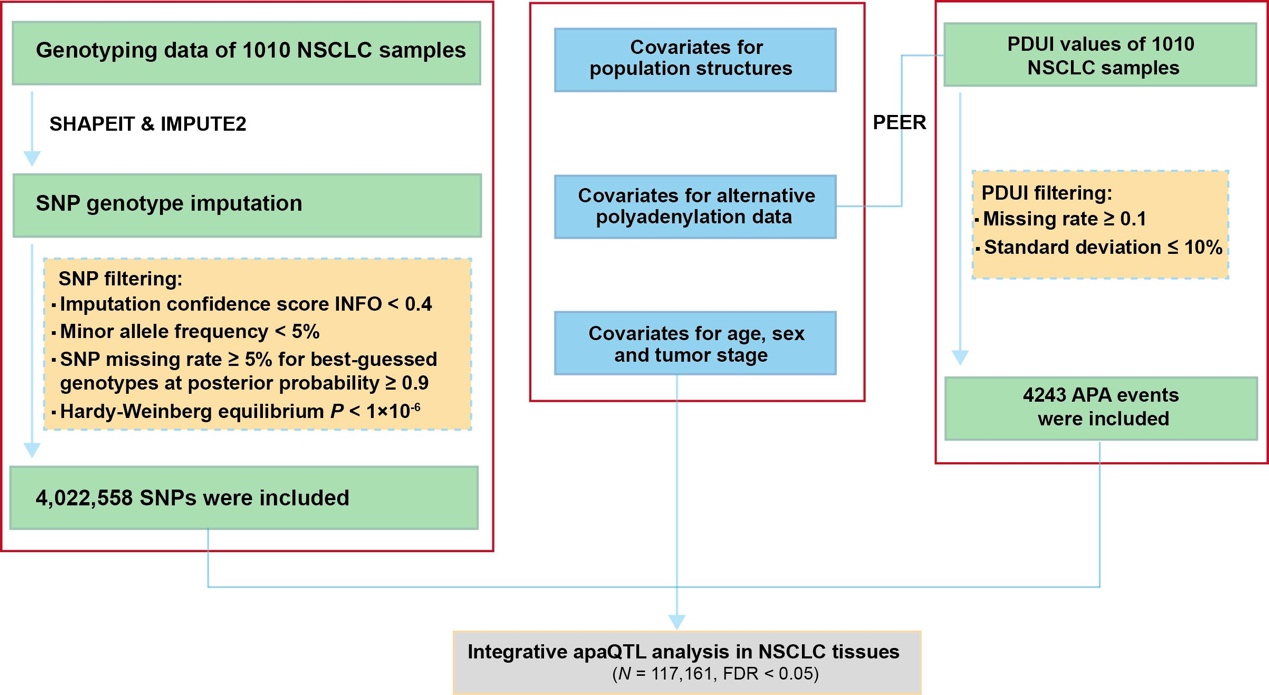


**Figure S1. Integrative apaQTL analysis in TCGA NSCLC tumor tissues.** The flowchart of integrative apaQTL analysis. A total of 117,161 apaQTLs were identified with FDR < 0.05.

**
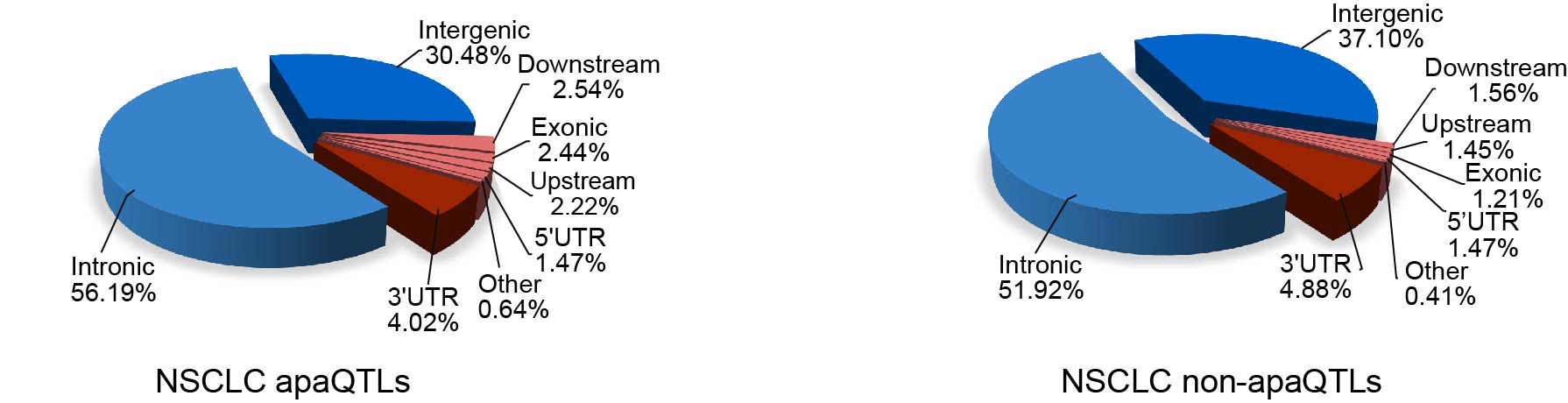
**

**Figure S2**. **Genomic distribution of apaQTLs identified in TCGA NSCLC samples**. Pie charts indicate the proportions of apaQTLs (left) and non-apaQTLs (right) in various genomic locations.


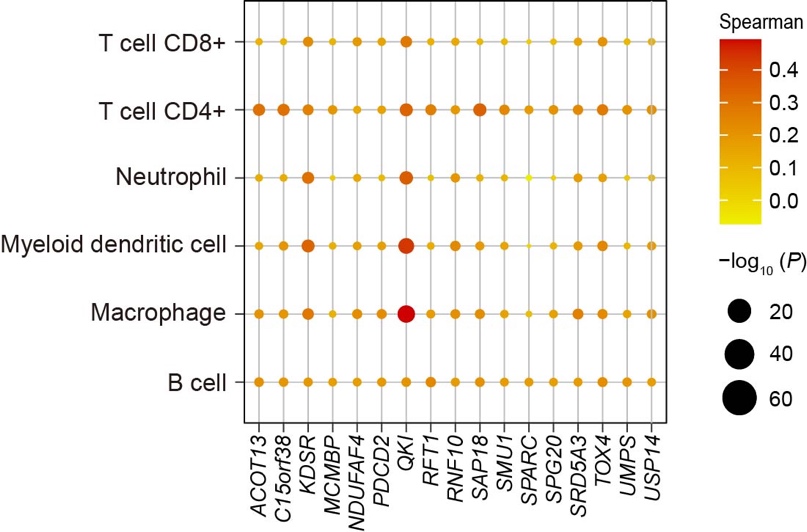


**Figure S3.** Bubble diagram showing the association between aGenes and individual immune cell infiltration in NSCLC. The color intensity indicates the Spearman correlation of immune cell fractions (rows) with aGene expression (columns). Circle size indicates -log_10_(*P*) value.

**Figure S4.** The association between the expression of aGenes and infiltration proportion of immune cells, in which the immune cell infiltration data were quantified using EPIC algorithm.
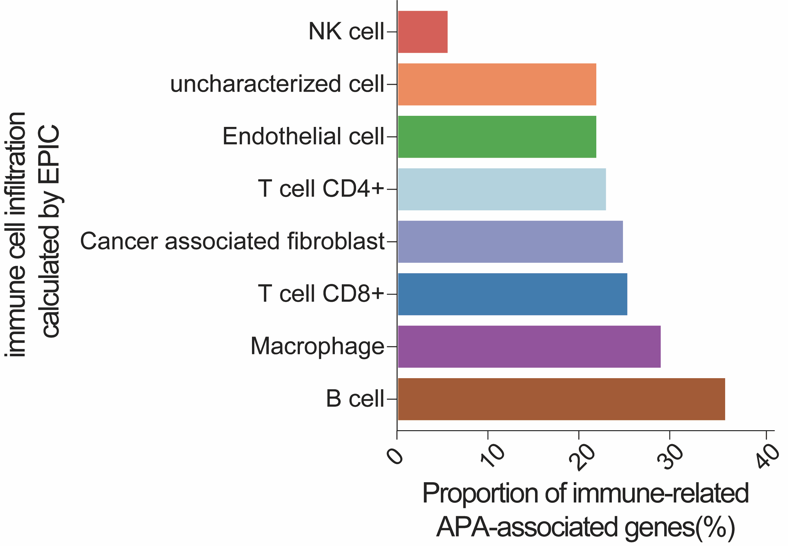


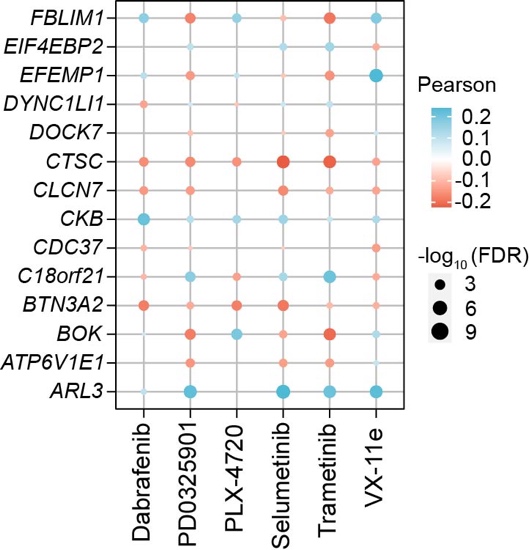


**Figure S5.** Detailed results of correlation of aGene expression with drug sensitivity that target ERK/MAPK signaling. The circle color represents the Pearson correlation, while the circle size represents -log_10_(FDR) value.

**
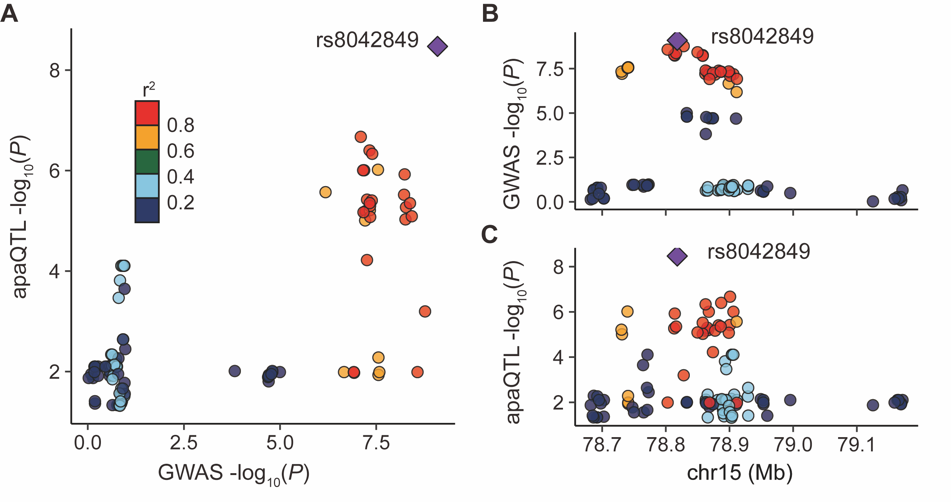
Figure S6.** Aligned Manhattan plots of apaQTLs colocalized with NSCLC GWAS variants at the 15q25.1 locus generated by LocusCompare (**A**). SNPs are colored by LD (r^2^) with the lead tRFQTL (rs8042849). The GWAS *P*-value of each SNP (**B**) and the apaQTL *P*-value of each SNP (**C**) are shown in the plot.

**Figure S7.** Proportion of NSCLC heritability explained by apaQTLs (red) and eQTLs (green). Bars indicate standard errors. GWAS summary data were obtained from the Finnish biobank (FinnGen)
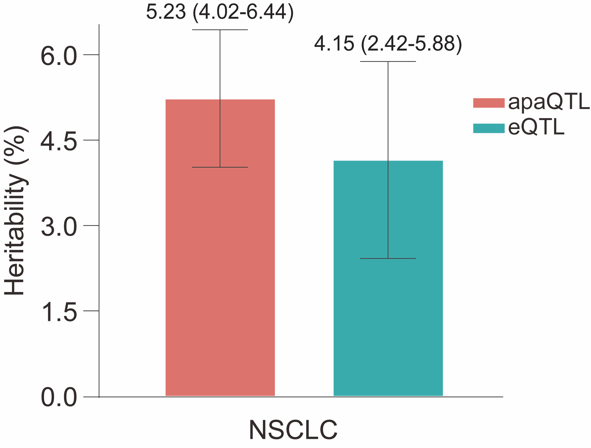
.


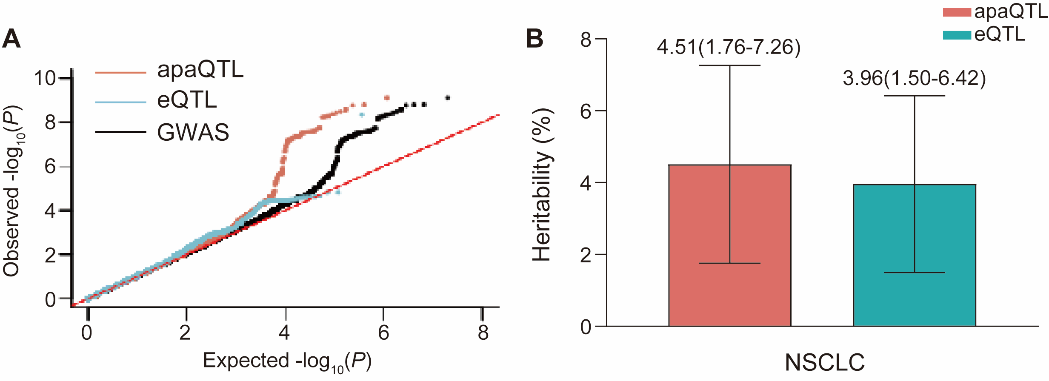


**Figure S8.** QQ plot analysis (A) and heritability estimation (B) after removing apaQTLs that overlapped with eQTLs.


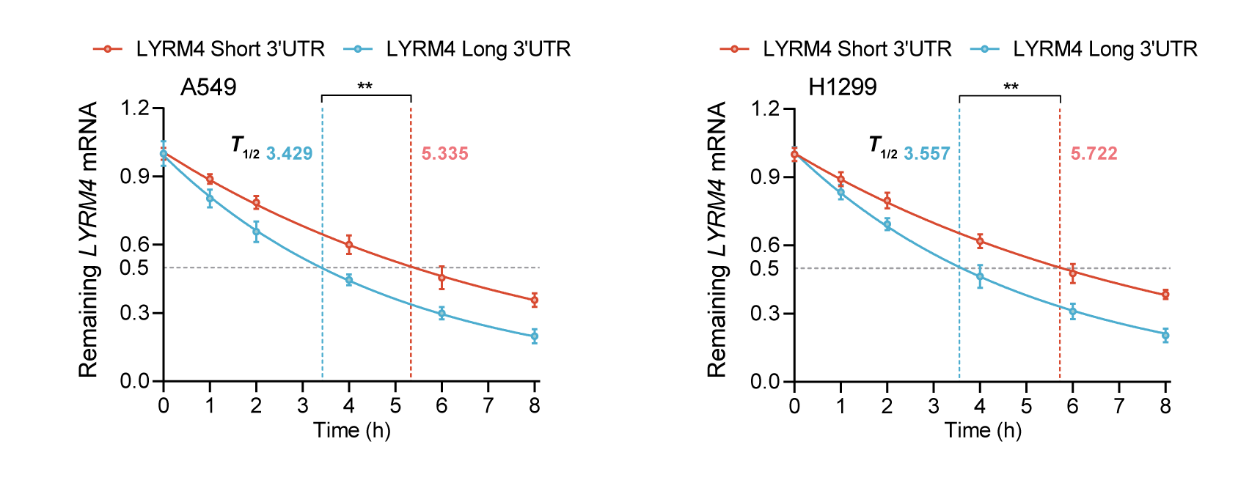


**Figure S9.** qRT-PCR was performed to evaluate the effect of LYRM4 3'UTR length on LYRM4 mRNA stability in A549 and H1299 cells after treating with actinomycin D (5μg/mL) at indicated time points.


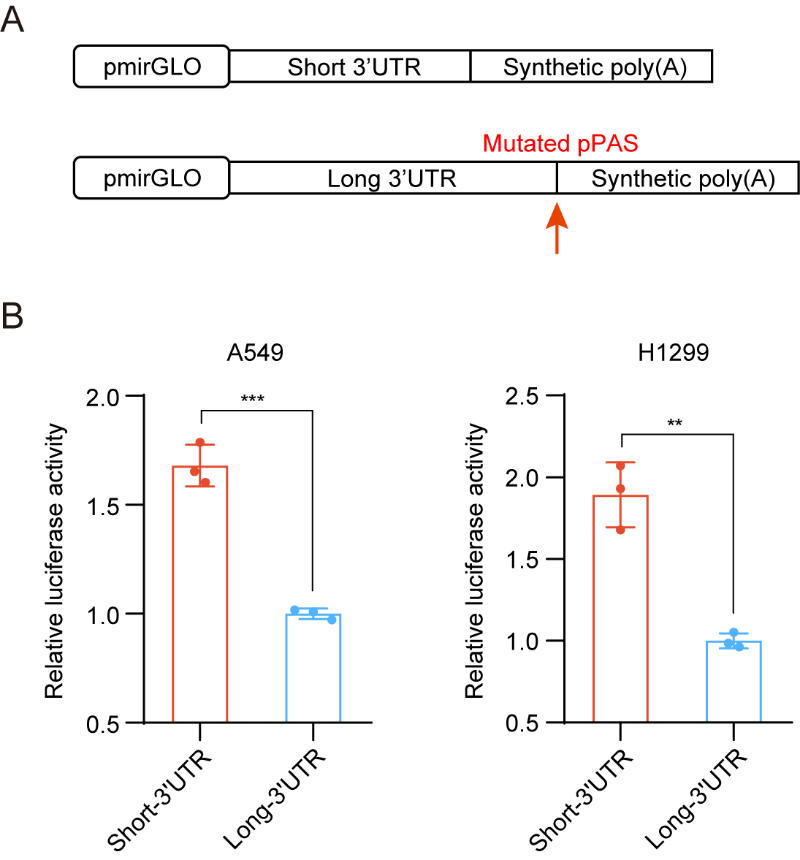


**Figure S10.** **A**, Schematic of the dual luciferase reporter plasmids construction. The red arrow indicates the mutated proximal polyadenylation signal. **B**, Luciferase activity in A549 and H1299 cells after transfection with the reporter plasmids containing the short and long 3'UTR of *LYRM4*. Results were shown as mean ± SD of three independent experiments. ***P* < 0.01 and ****P* < 0.001 were calculated using Student’s *t* test.


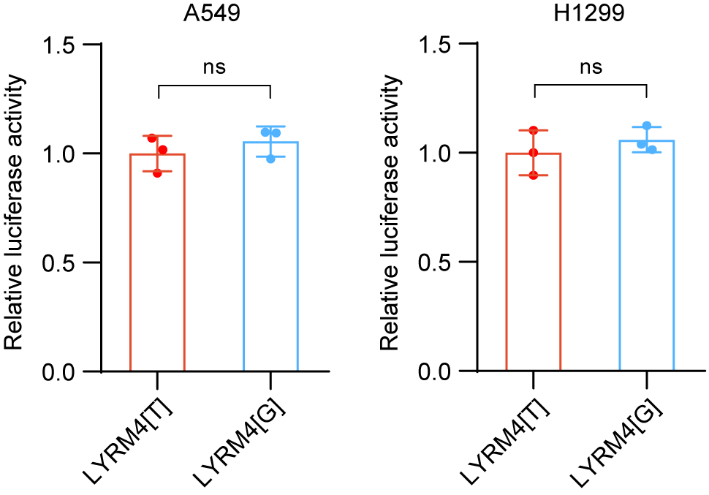


**Figure S11.** Luciferase activity in A549 and H1299 cells after transfection with the reporter plasmids containing the rs9606 T allele or G allele. Results were shown as mean ± SD of three independent experiments. No significant difference (n.s.) was calculated using Student’s t test.


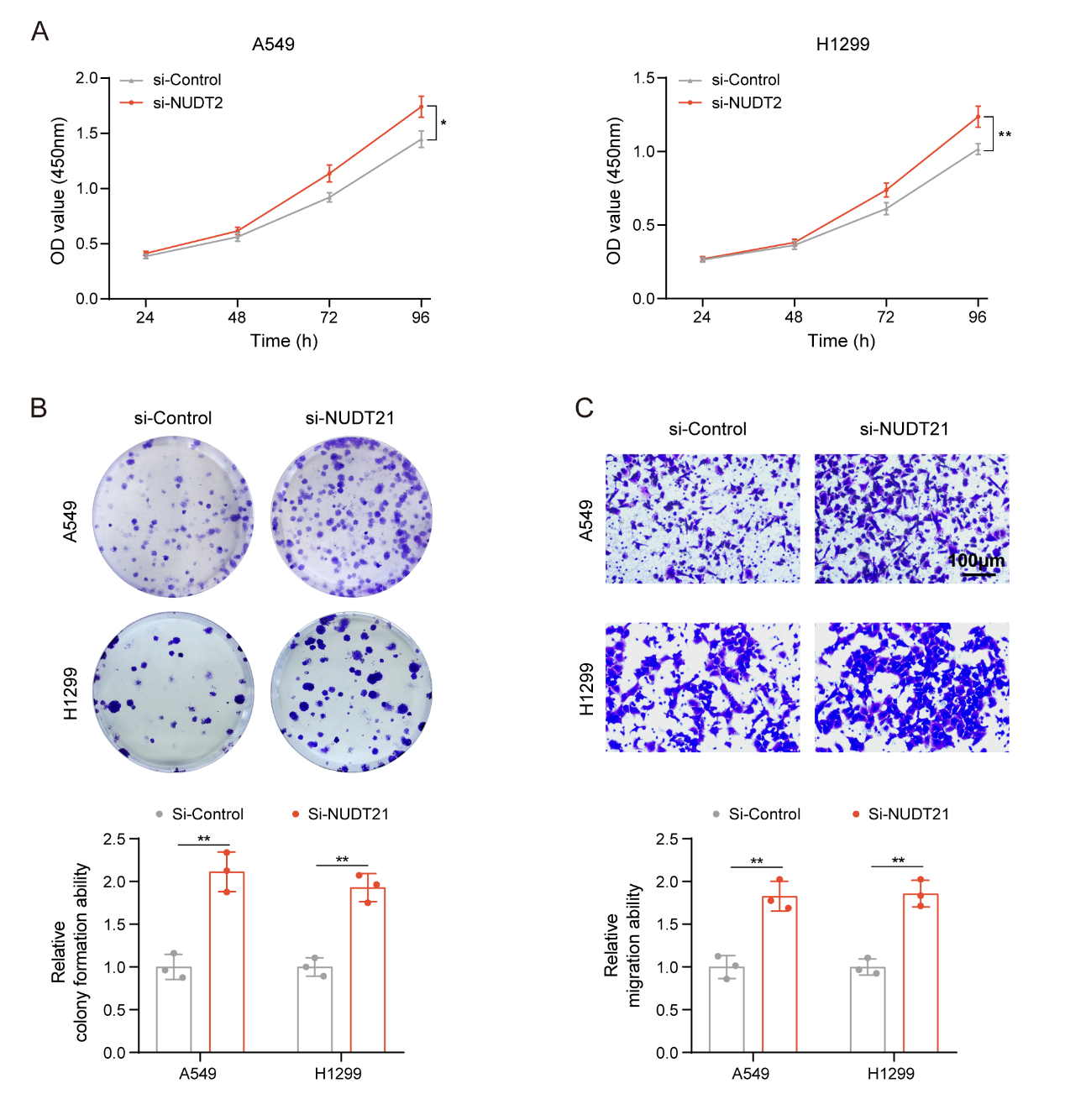


**Figure S12. *NUDT21* knockdown promotes NSCLC cell proliferation and migration. A**, The effect of *NUDT21* knockdown on cell proliferation determined by CCK-8 assays. **B**, The effect of *NUDT21* knockdown on colony formation ability of NSCLC cells. **C**, The effect of *NUDT21* knockdown on migration ability of NSCLC cells in vitro. Results were shown as the mean ± SD from three independent experiments. **P* < 0.05 and ***P* < 0.01were calculated using Student’s t test.


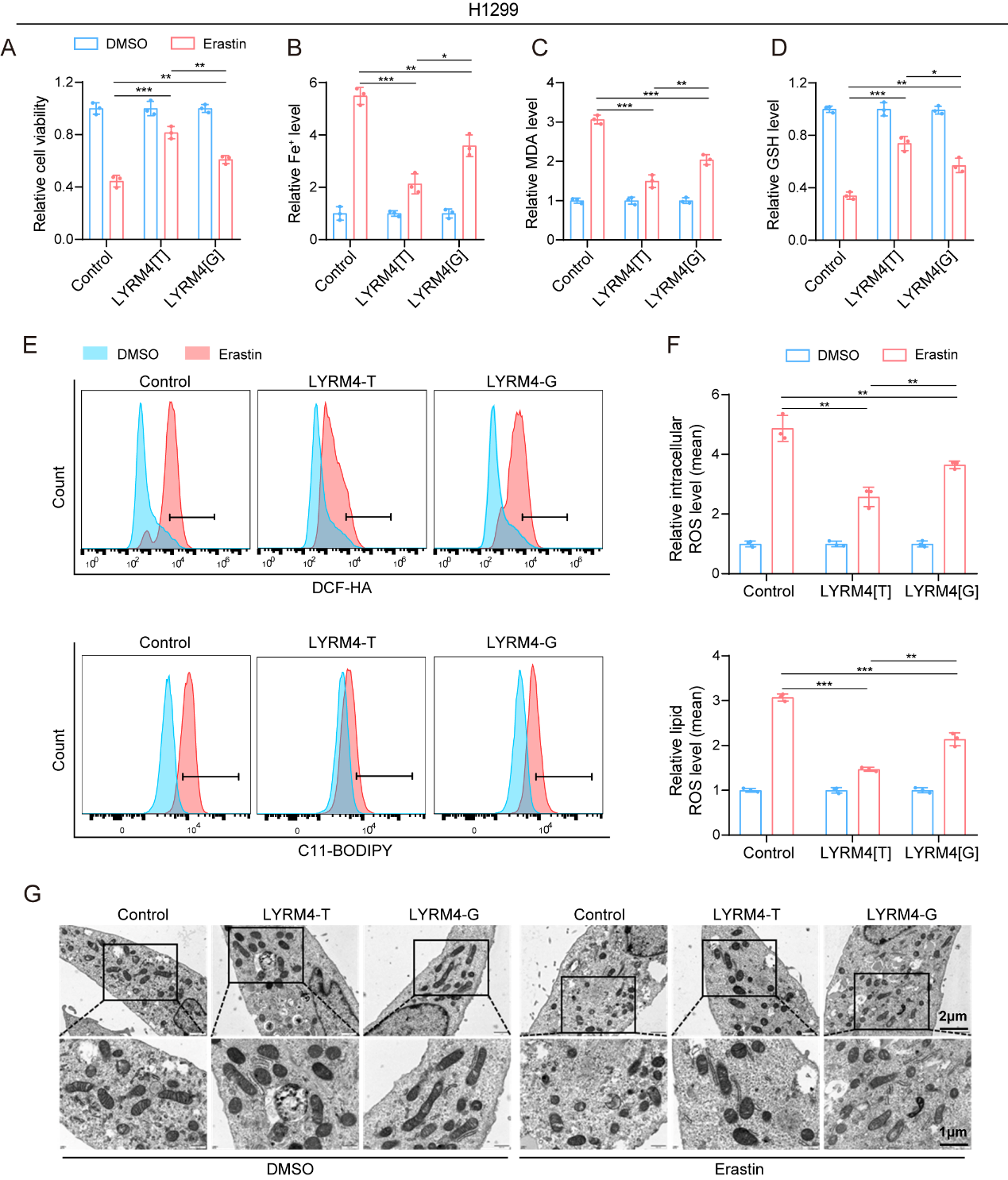


**Figure S13.** **Risk T allele at *LYRM4* suppressing ferroptosis activity of H1299 cells. A-D,** Cell viability (**A**), Fe^2+^ levels (**B**), MDA levels (**C**) and GSH levels (**D**) were measured. H1299 cells were transfected LYRM4[T]**,** LYRM4[G], and control vector. After transfection, cells were treated with DMSO or erastin (10 μM) for 24 h. Results were shown as the mean ± SD from three independent experiments. **E** and **F**, Intracellular ROS and lipid ROS levels were detected using flow cytometry with DCFH-DA and BODIPY staining, respectively. H1299 cells were transfected with indicated DNA constructs. After transfection, cells were treated with DMSO or erastin (5 μM) for 24 h. Representative images (**E**) and quantitative static results (**F**) were shown. Results were presented as the mean ± SD from three independent experiments. **G,** Morphological changes of mitochondria were detected by transmission electron microscopy. H1299 cells were transfected with indicated DNA constructs, followed by treatment with DMSO or erastin (10 μM) for 24 h. *P* values were calculated using Student’s *t* test. *, *P* < 0.05; **, *P* < 0.01; ***, *P* < 0.001.
